# Supplementary material for: Ancient exapted transposable elements promote nuclear enrichment of human long noncoding RNAs
Source: Genome Res. 2019 Feb;29(2):208–22. doi: 10.1101/gr.229922.117 (PMC6360812; doi:10.1101/gr.229922.117)
Supplement: Supplemental Material [file supp_29_2_208__index.html]

Ancient exapted transposable elements promote nuclear enrichment of human long noncoding RNAs — Ancient exapted transposable elements promote nuclear enrichment of human long noncoding RNAs — Supplemental Material 

# Ancient exapted transposable elements promote nuclear enrichment of human long noncoding RNAs

## Supplemental Material

- Supplemental\_Figures.pdf
- Supplemental\_File\_S1.bed
- Supplemental\_File\_S2.pdf
- Supplemental\_File\_S3.txt
- Supplemental\_File\_S4.docx
- Supplemental\_File\_S5.bed
- Supplemental\_File\_S6.docx
- Supplemental\_File\_S7.docx
- Supplemental\_Legends.pdf
- Supplemental\_Code.pl.zip
