# Supplementary material for: Ancient exapted transposable elements promote nuclear enrichment of human long noncoding RNAs
Source: Genome Res. 2019 Feb;29(2):208–22. doi: 10.1101/gr.229922.117 (PMC6360812; doi:10.1101/gr.229922.117)
Supplement: Supplemental Material [file supp_gr.229922.117_Supplemental_Figures.pdf]

Supplemental Figure S1

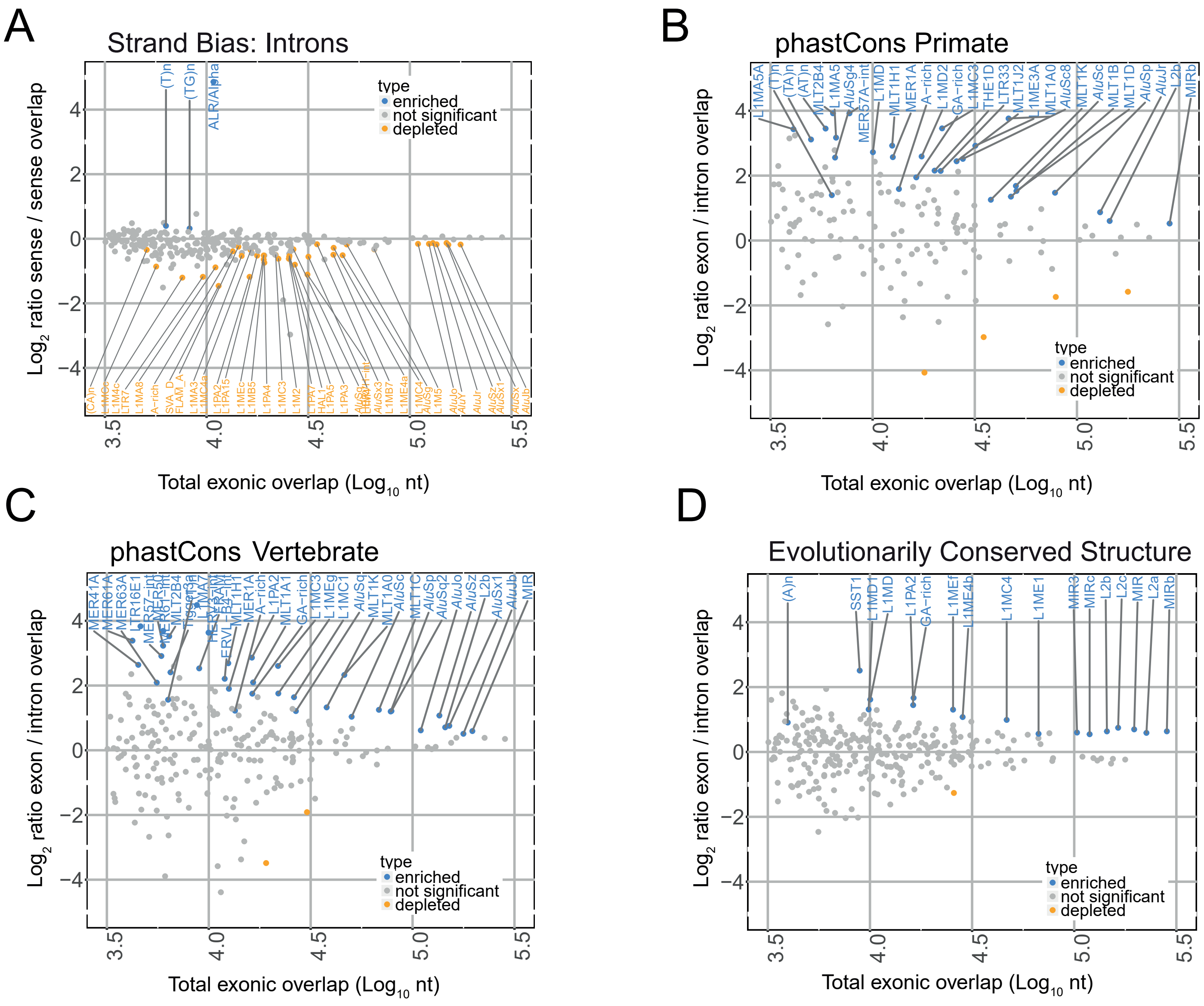

Supplemental Figure S1: (A) Figure shows, for every TE type, the ratio of intronic nucleotide coverage in sense vs antisense configuration. "Sense" here is defined as sense of TE annotation relative to the sense of the overlapping intron. Significantly-enriched TE types are shown in blue. Statistical significance was estimated by a randomisation procedure, and significance is defined at an uncorrected empirical p-value < 0.001 (See Material and Methods). (B-D) As for (A), but *y* axis records the ratio of nucleotide overlap in exons vs introns by phastCons primate-conserved elements, phastCons vertebrate-conserved elements and Evolutionarily Conserved Structures (ECS), respectively.

Supplemental Figure S2

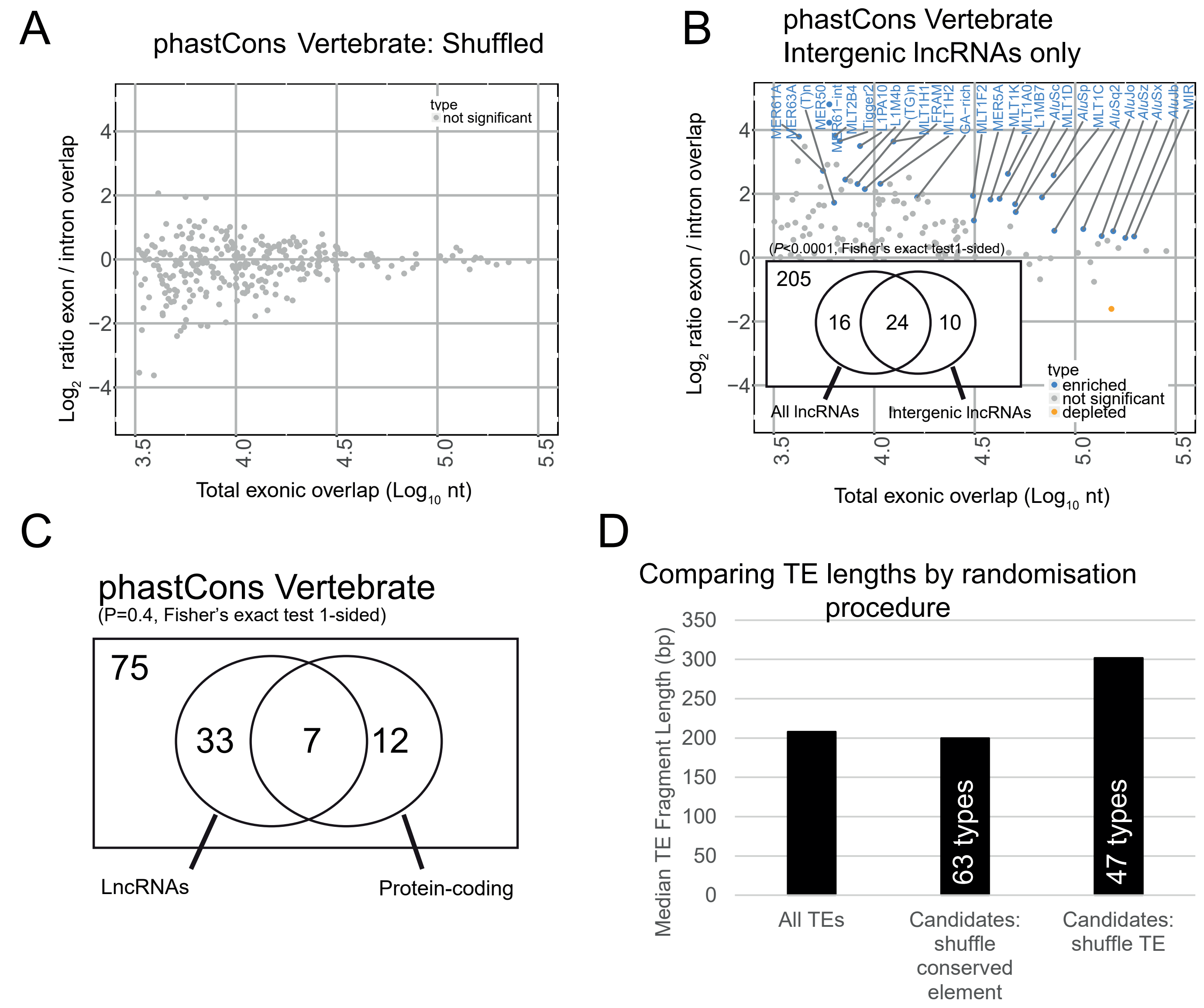

Supplemental Figure S2: (A) Figure shows, for every TE type, the ratio of exonic/intronic relative overlap by a shuffled set of phastCons Vertebrate elements. Significance was estimated by randomisation as in Figure 3, and here no significant TEs were identified. (B) Similar to (A), except that overlaps were performed using true phastCons Vertebrate conserved elements, and only the subset of intergenic lncRNA gene loci were included, ie those not overlapping a protein-coding locus. Blue indicates enriched TE types, as estimated by randomisation with an uncorrected *p*-value < 0.001. The inset shows the overlap of significant TE types between this analysis (right side) and the equivalent analysis using unfiltered lncRNAs (left, Supplemental Figure 1C). (C) Comparison of candidate RIDLs in lncRNAs and protein-coding genes, identified by overlap with phastCons Vertebrate elements. Numbers indicate TE types. (D) Comparison of lengths of significantly-conserved, exonically-overlapping TEs identified by two different randomisation procedures: shuffling conserved region locations, holding TEs constant (method used in Results); shuffling TE locations, holding conserved regions constant. For comparison, the lengths of the entire set of exonic TEs used in the study are shown ("All TEs"). Note that lengths are calculated using the entire TE fragment, and not just the portion overlapping the exon.

Supplemental Figure S3

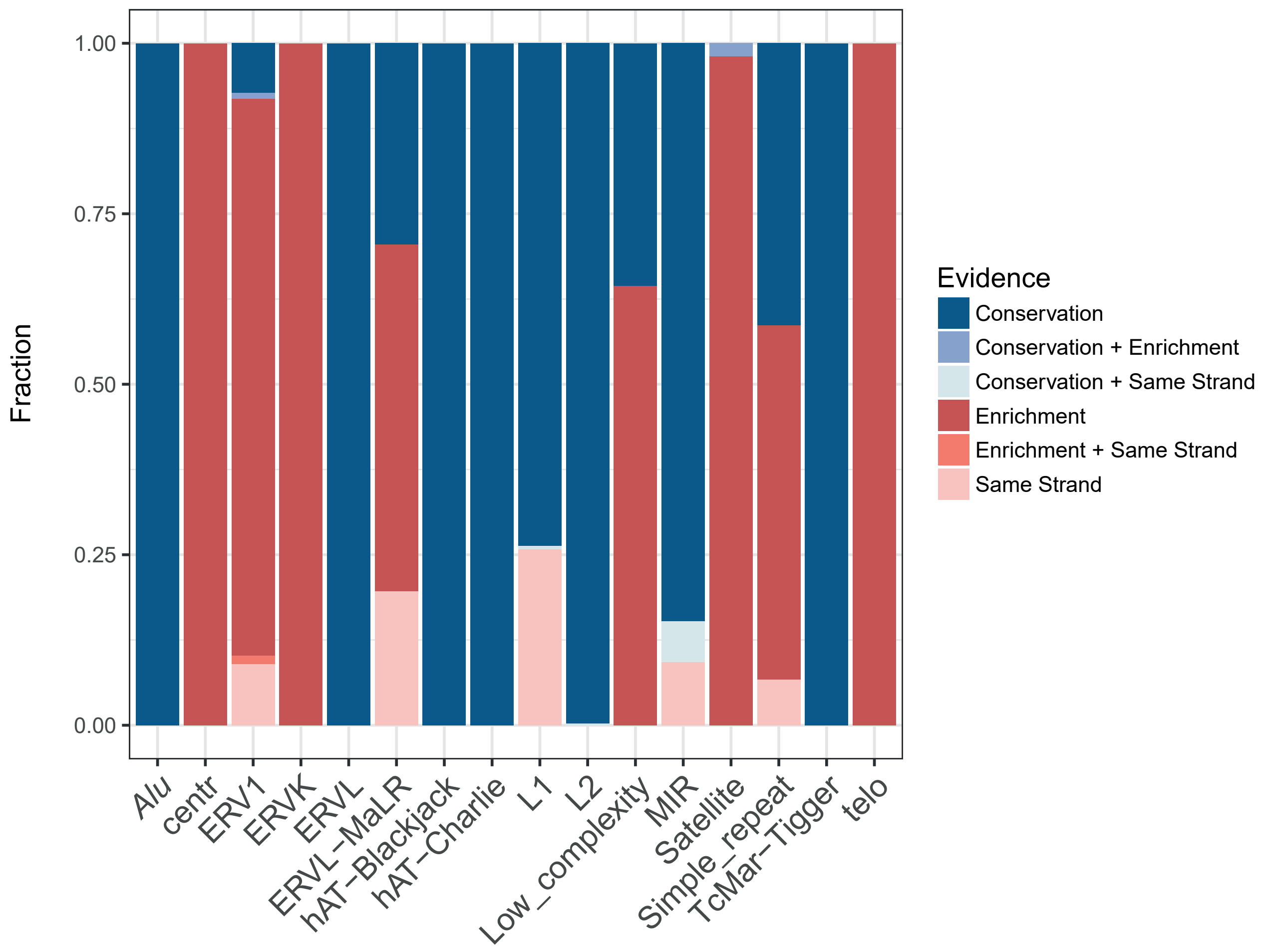

Supplemental Figure S3: Proportional frequencies of RIDLs identified by evidence type.

Supplemental Figure S4

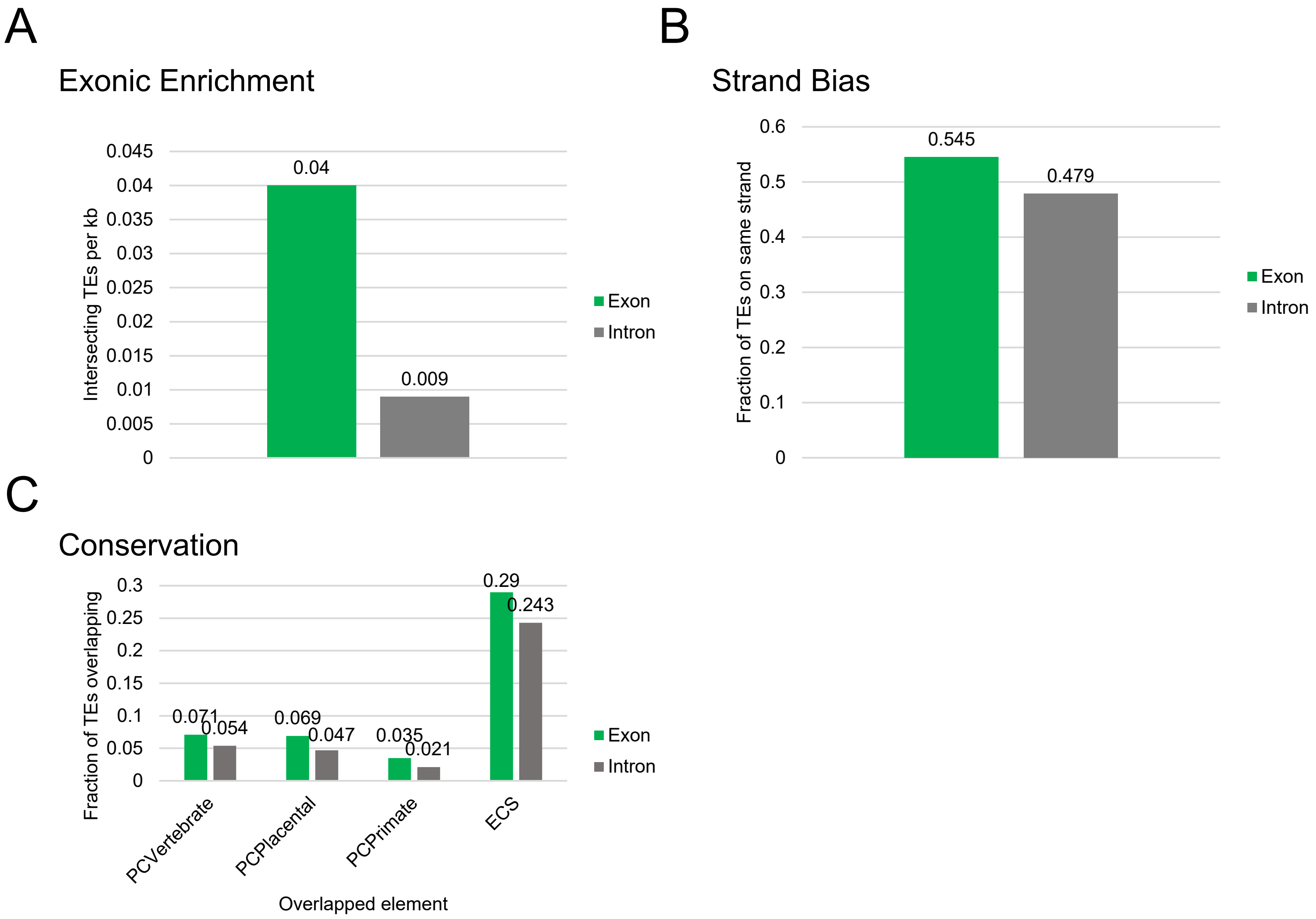

Supplemental Figure S4: Estimating true positive rates for RIDL predictions. Figures show fractions of TEs broken down by exonic and intronic cases. Only TEs from significant types for each evidence source, as outlined in Materials and Methods and shown in Figure D, are considered. We interpret the value for introns to represent an upper limit of the false discovery rate, and the difference between exon and intron to represent the number of true positive predictions. (A) The number of TEs from 20 types defined to be exonically enriched, which overlap either exons or introns of lncRNAs. (B) For RIDLs defined to have strand bias, the fraction of instances lying on the same strand as host lncRNA. (C) For RIDLs with evidence of evolutionary conservation, the fraction of instances that overlap the indicated conserved elements.

Supplemental Figure S5

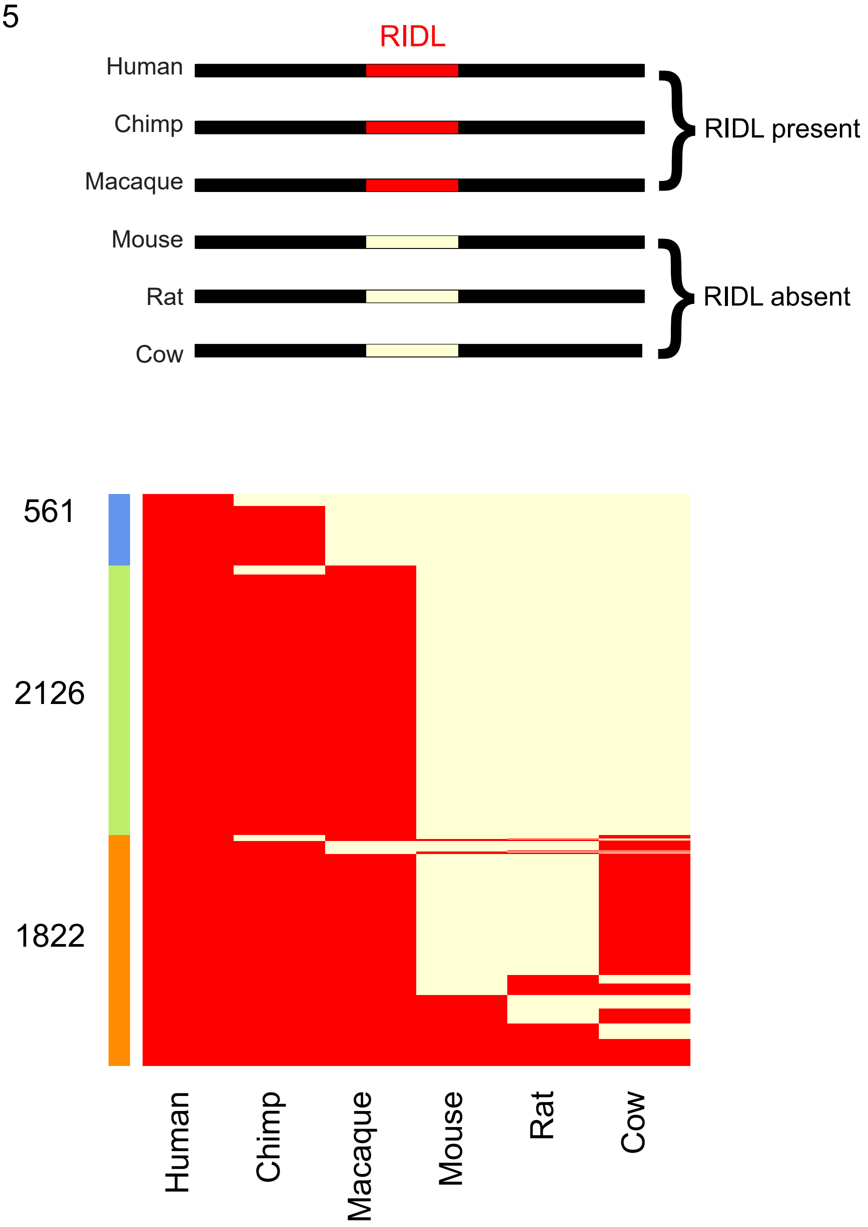

Supplemental Figure S5: Inferring the evolutionary age of RIDLs using 6-mammal alignments. Rows represent human RIDLs, columns represent species. Cells are coloured red when an orthologue is detected, otherwise in light yellow. Numbers represent the count of RIDLs conserved amongst Great Apes (blue), Primates (green) and Mammals (orange).

Supplemental Figure S6

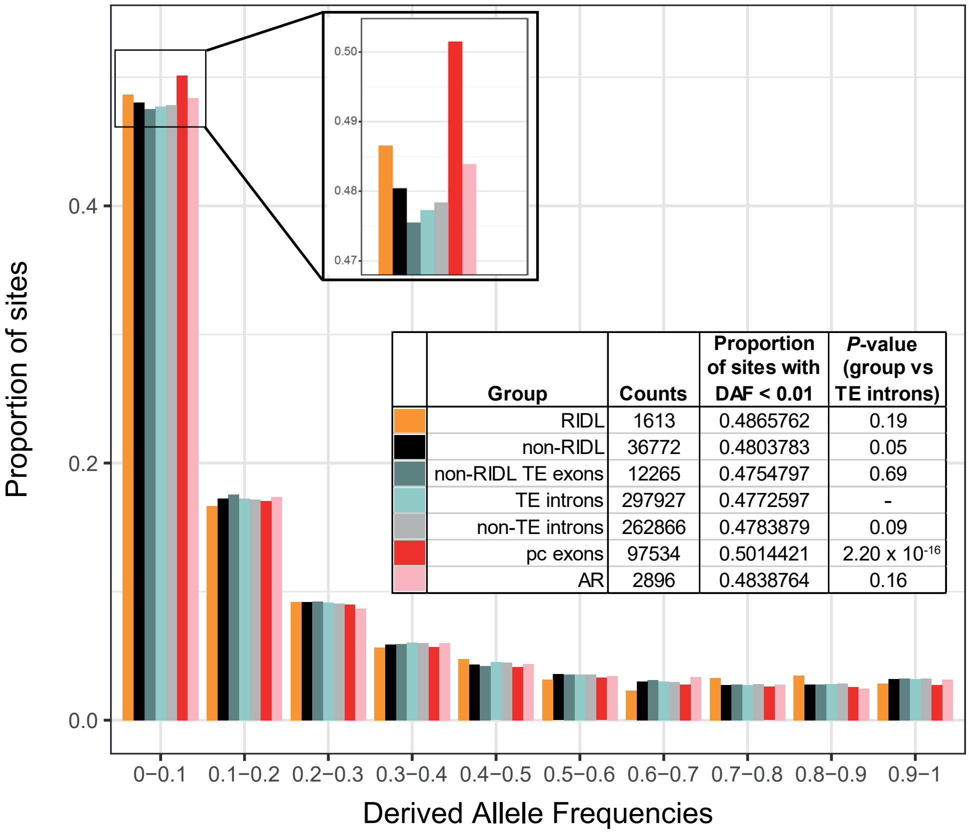

Group	Counts	Proportion of sites with DAF < 0.01	*P*-value (group vs TE introns)
RIDL	1613	0.4865762	0.19
non-RIDL	36772	0.4803783	0.05
non-RIDL TE exons	12265	0.4754797	0.69
TE introns	297927	0.4772597	-
non-TE introns	262866	0.4783879	0.09
pc exons	97534	0.5014421	2.20×10^{-16}
AR	2896	0.4838764	0.16

Supplemental Figure S6: Proportion of common SNPs falling in the different bins of derived allele frequencies (DAF) for every group described in the table (left column). Protein coding exons (pc exons) and ancestral repeats (AR) are shown as positive and negative controls, respectively. The table shows for every group the total SNPs counts (second column) with low-frequency alleles (DAF < 0.1), the proportion this represents (third column) and the *p*-value obtained from comparing each group to TE introns (taken as a background) (Fisher's exact test, 1-sided).

Supplemental Figure S7

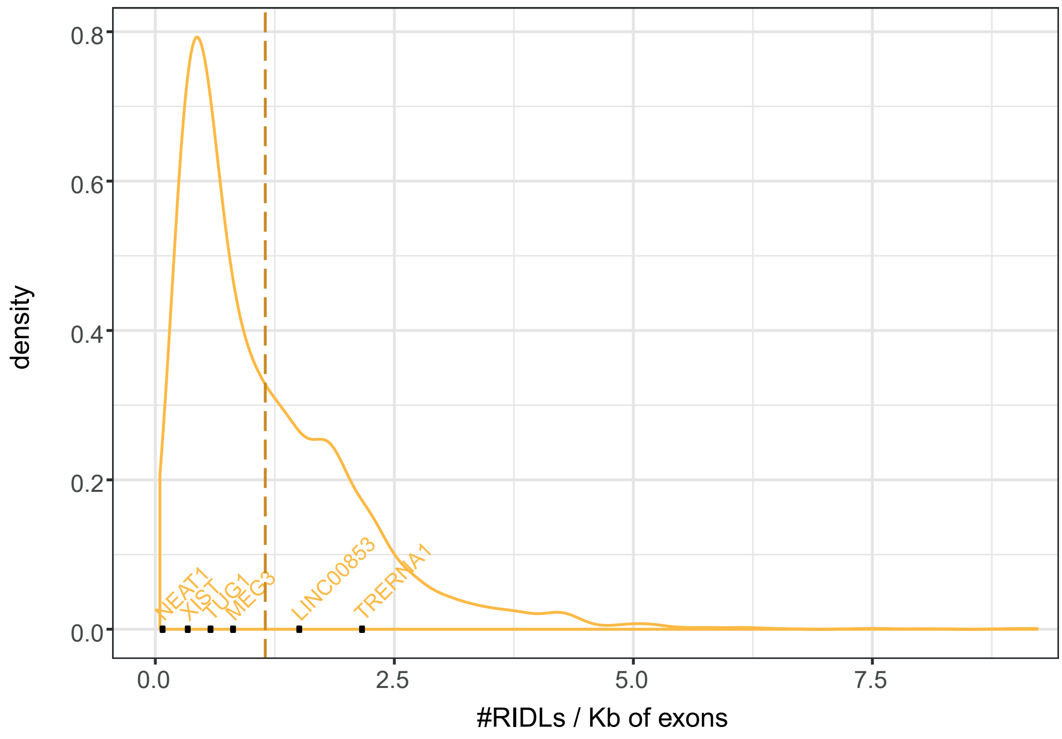

Supplemental Figure S7: Density distribution of RIDL-carrying lncRNAs based on the number of RIDLs per kb of exon. Dotted line indicates median. Selected known lncRNAs are indicated.

Supplemental Figure S8

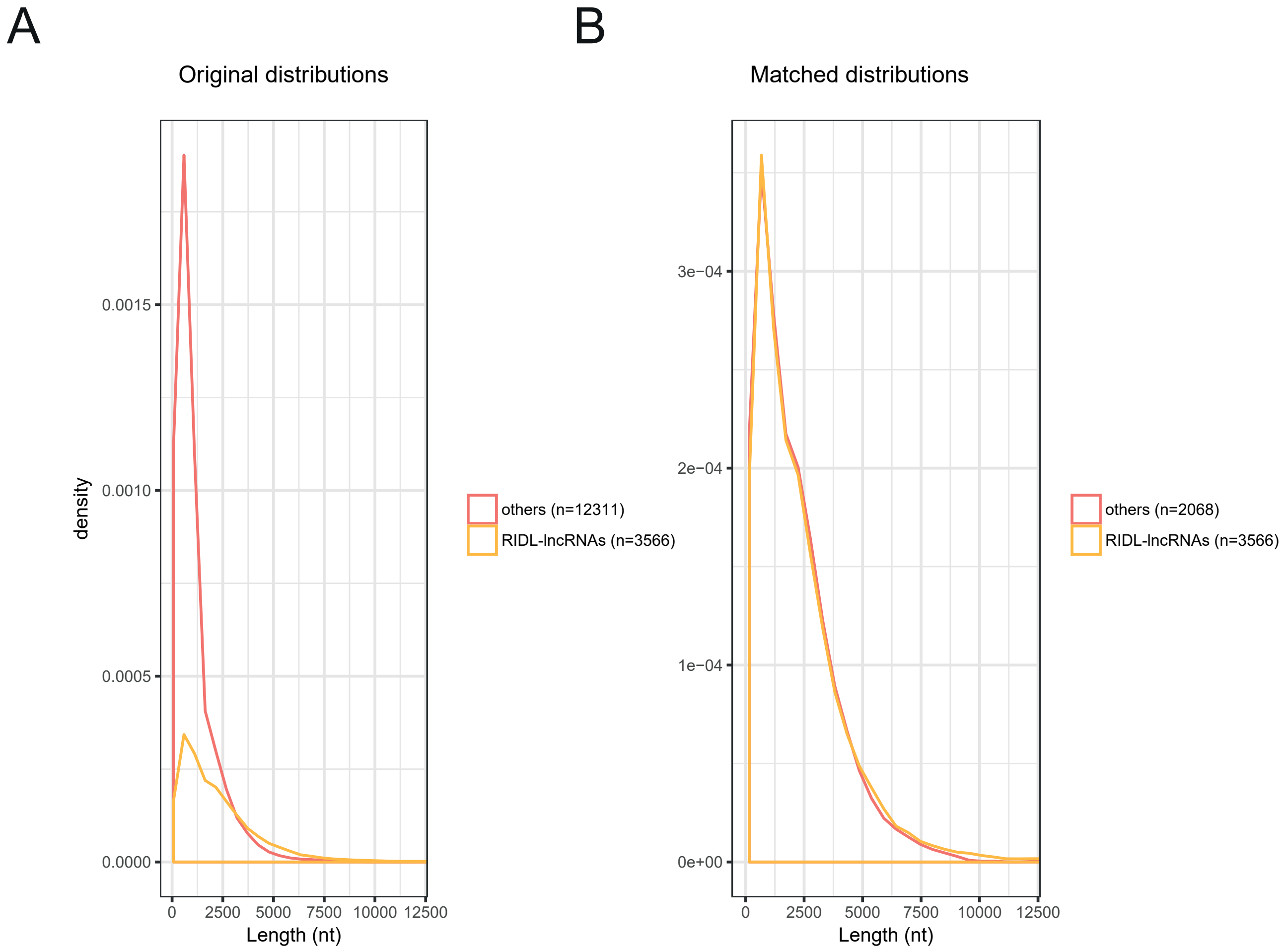

Supplemental Figure S8: Creating a length-matched sample of non-RIDL lncRNAs. (A) Exonic length distribution of RIDL-carrying (RIDL-lncRNAs) and non-RIDL ("others") GENCODE v21 lncRNAs, prior to sampling. (B) Exonic length distribution of RIDL-carrying lncRNAs and a length-matched sample of non-RIDL lncRNAs. The latter were used for all comparisons at gene level.

Supplemental Figure S9

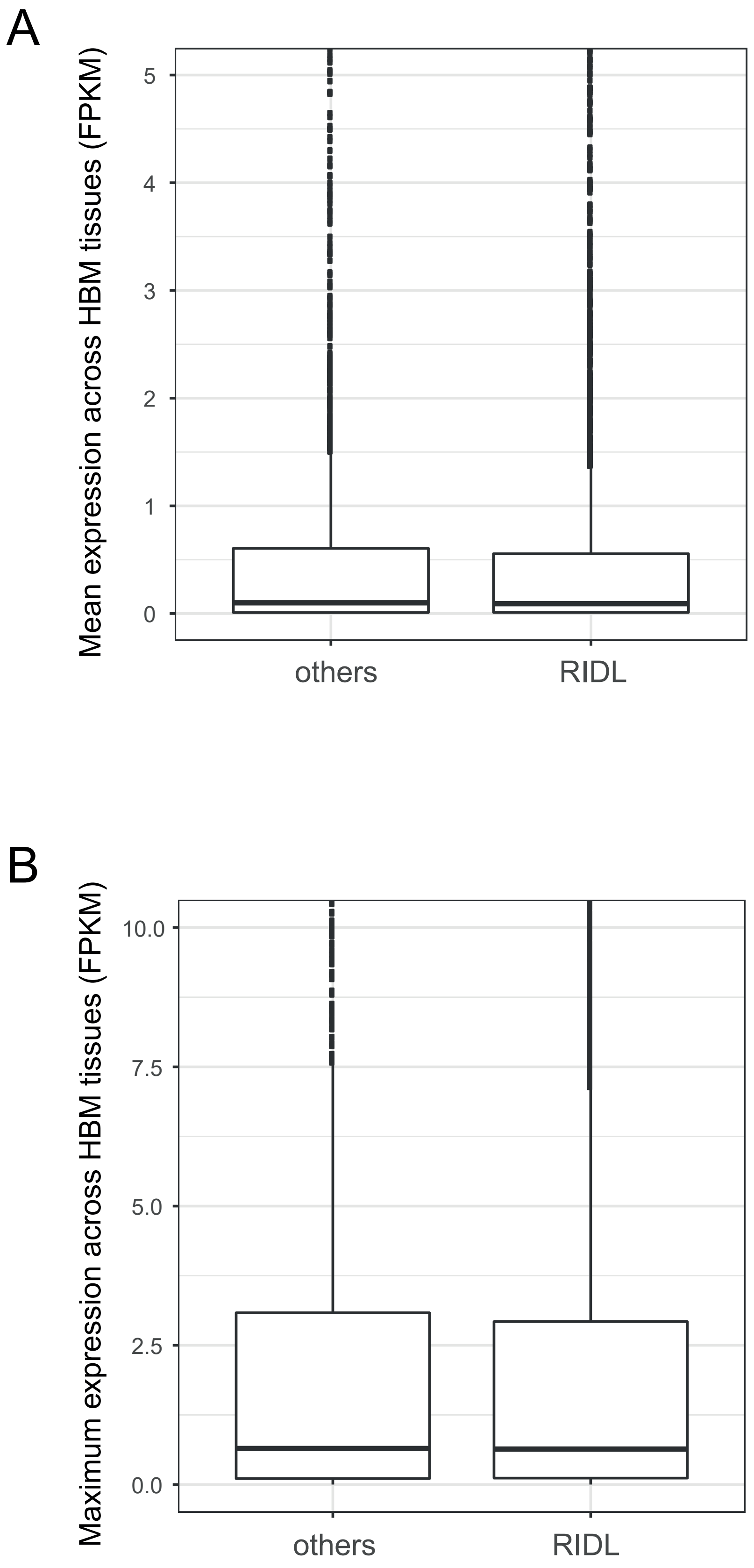

Supplemental Figure S9: (A) Distribution of mean expression across Human Body Map (HBM) tissues of RIDL-lncRNAs and other length-matched lncRNAs (others). (B) Same as (A) for maximum expression across HBM tissues.

Supplemental Figure S10

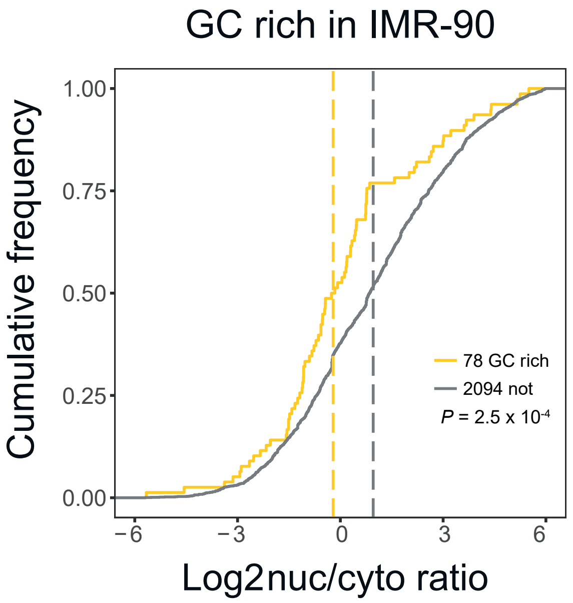

Supplemental Figure S10: Nuclear/cytoplasmic localization of GC-rich RIDL-containing lncRNAs in IMR-90. Yellow represents lncRNAs carrying one or more copies of GC-rich RIDL elements, grey represents all other detected lncRNAs ("not"). Dashed lines indicate the median of each group. Significance was calculated using Wilcoxon test (*P*).

Supplemental Figure S11

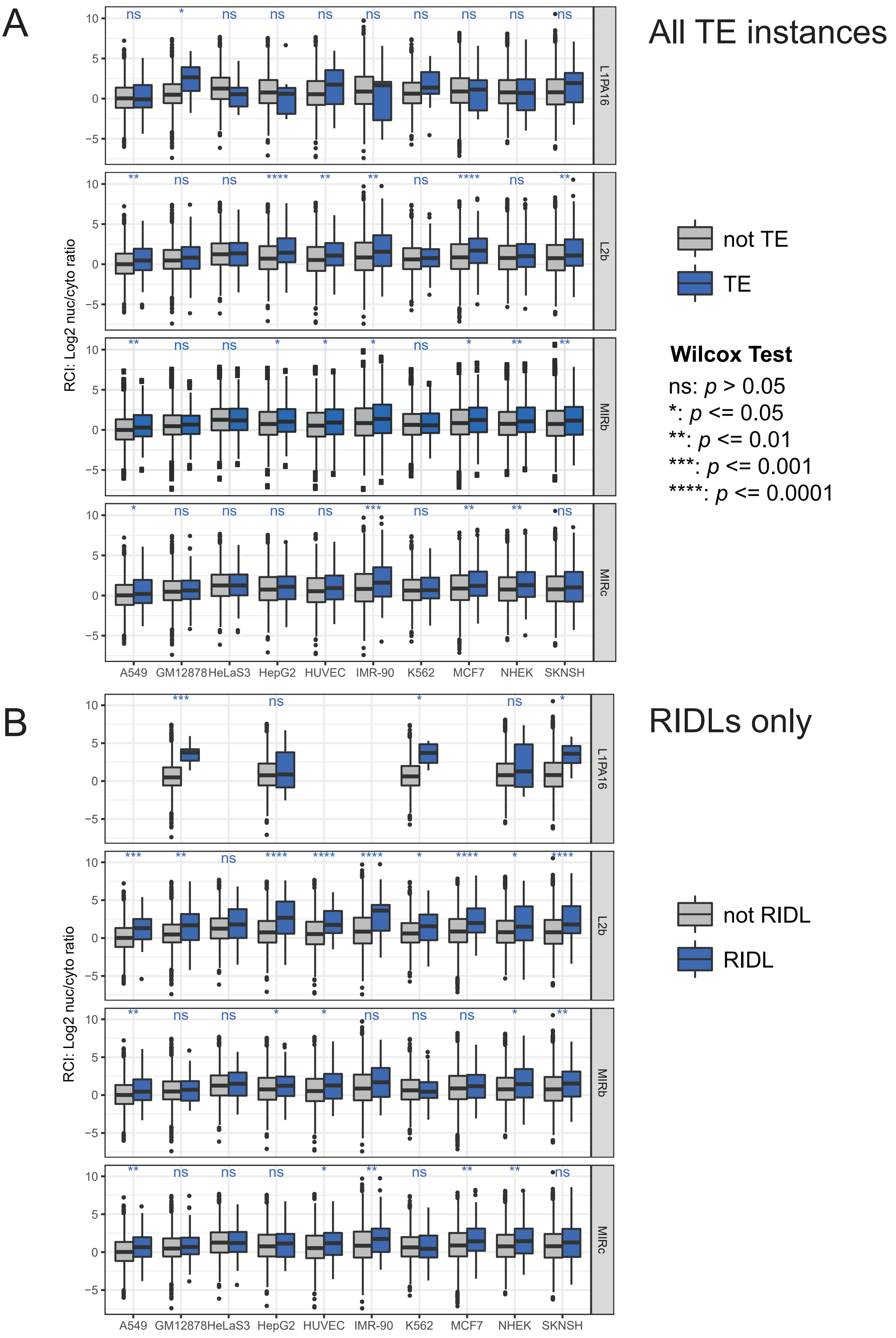

Supplemental Figure S11: Presence of L1PA16, L2b, MIRb and MIRc in lncRNA exons correlate with localisation, when considering both unfiltered TEs and RIDL sets. (A) For every cell line boxplots show RCI values of unfiltered TE-carrying lncRNAs versus non-TE-carrying lncRNAs (each panel contains information for a different TE type). (B) As for (A) but comparing RIDL-lncRNAs vs non-RIDL-lncRNAs.

Supplemental Figure S12

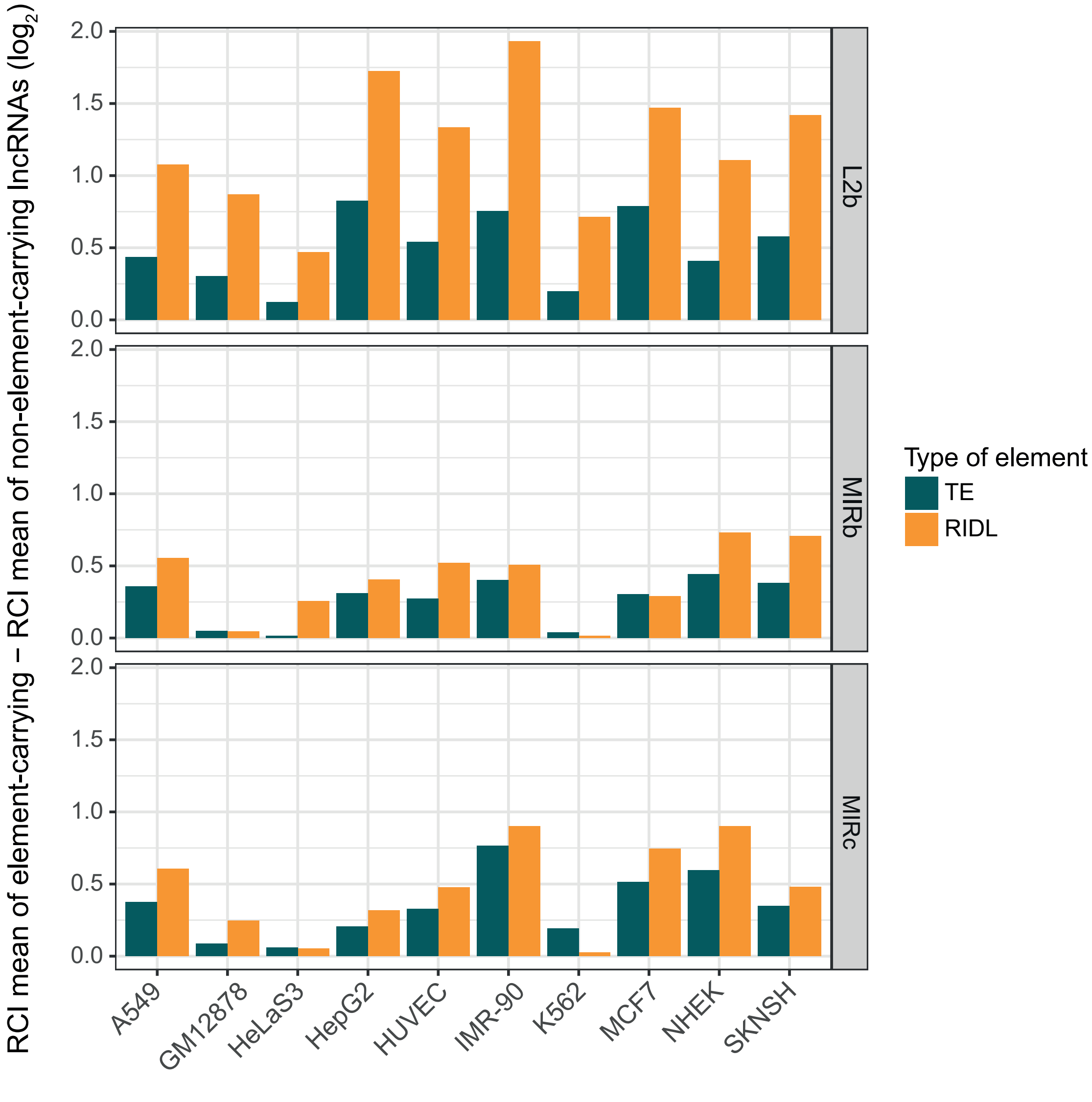

Supplemental Figure S12: RIDLs have a stronger effect on localisation than unfiltered TEs. Bars indicate the log_2 difference between the RCI (nuc/cyto ratio) of element-carrying lncRNAs and non-TE lncRNA.

Supplemental Figure S13

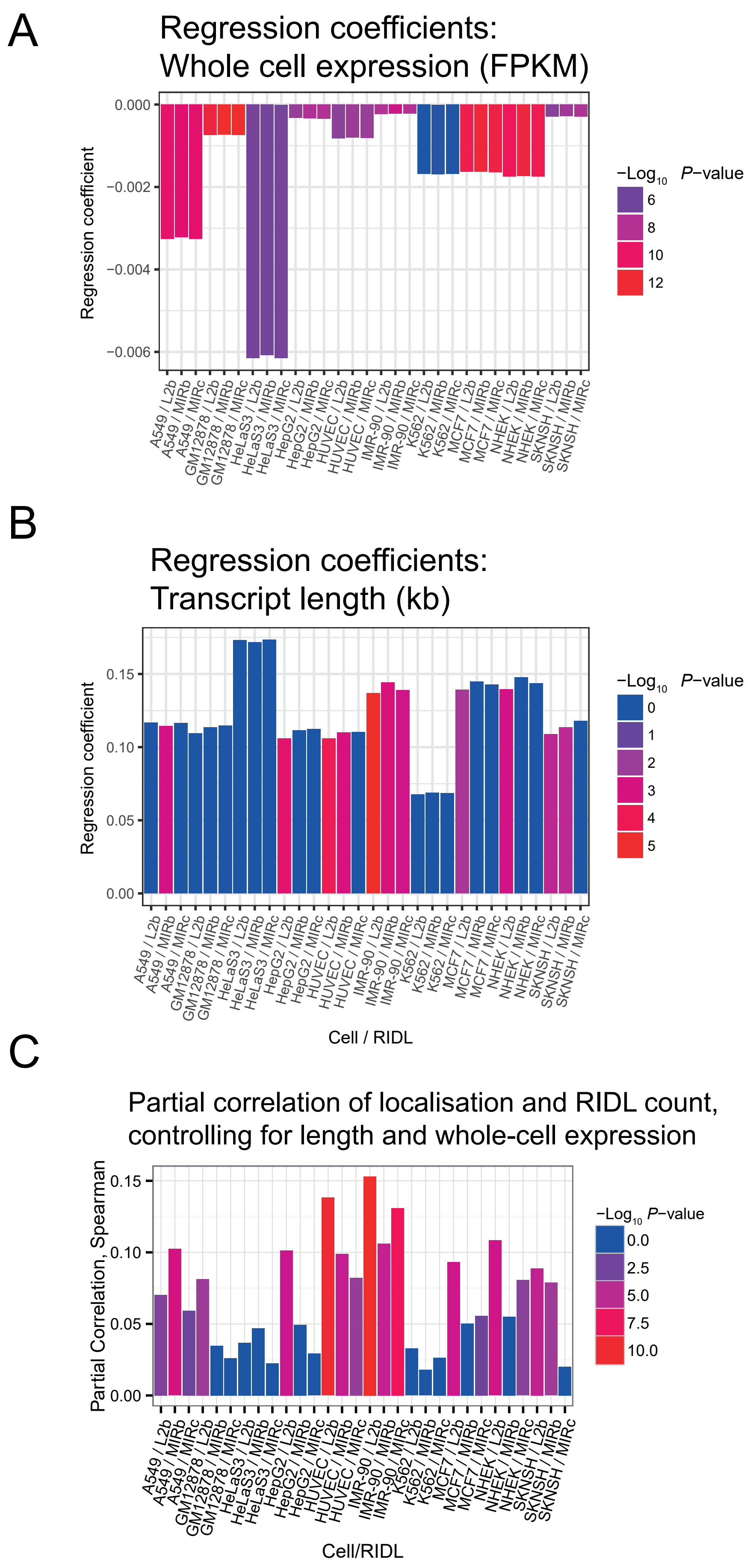

Supplemental Figure S13: (A) Regression coefficients for explanatory variable of whole-cell expression (in units of FPKM), in a linear model where dependent variable is lncRNA nuclear/cytoplasmic localisation (see Figure 5E and Methods). *x*-axis represents each RIDL/cell-line combination. Colours reflect estimated *p*-value for the association. (B) As for (A), but for explanatory variable of transcript length (in kb). (C) Partial correlation coefficients (Spearman) of $\log_2$ nuclear/cytoplasmic localisation and RIDL count, controlling for length and whole-cell expression. *x*-axis represents each RIDL/-cell-line combination. Colours reflect estimated *p*-value for the association.

Supplemental Figure S14

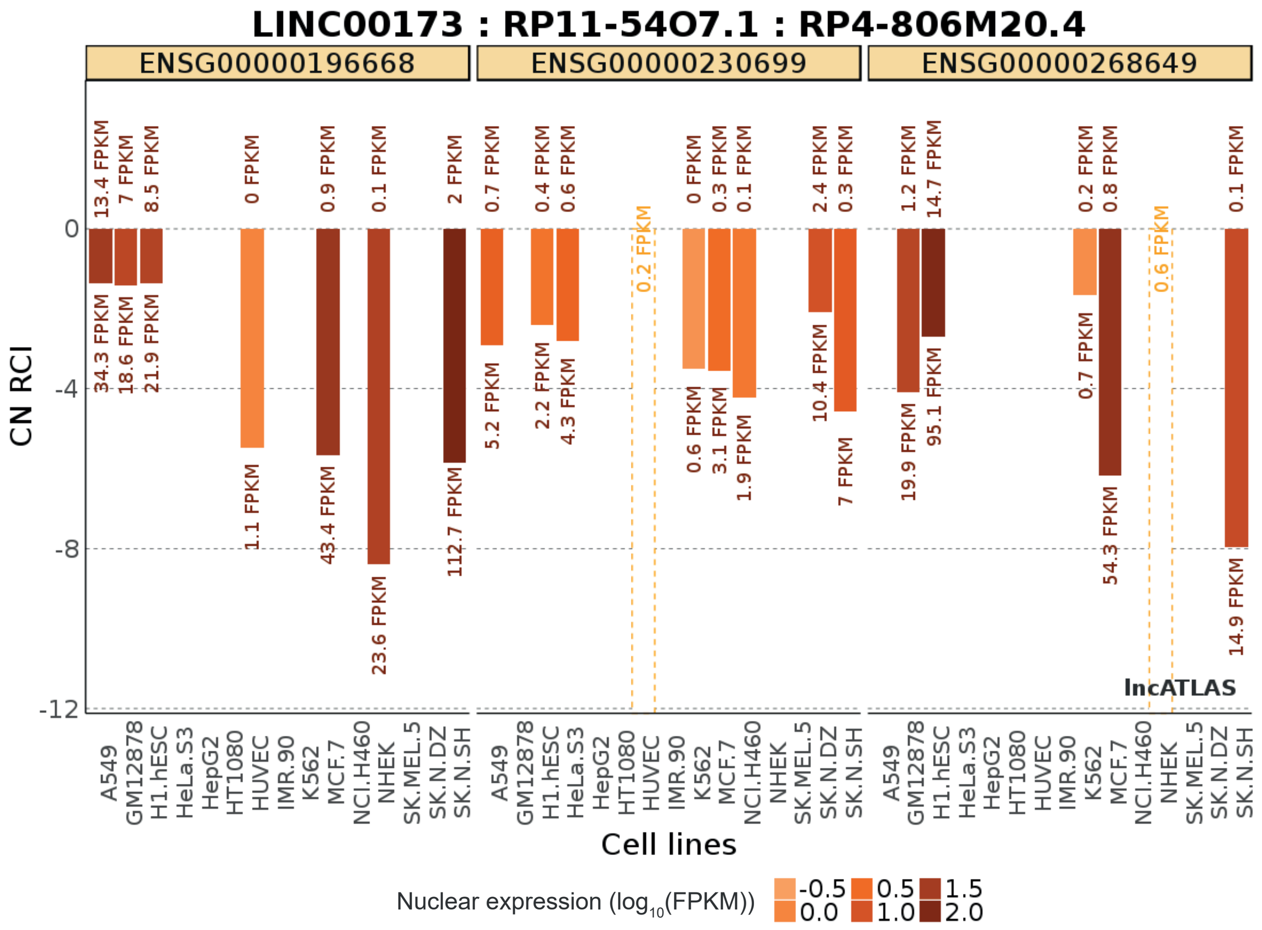

Supplemental Figure S14: Bars indicate log_2-transformed cytoplasmic/nuclear expression ratios (CN RCI) in 15 cell lines for three candidate genes. Colours represent the total nuclear expression of each gene in each cell line. Barplot generated by LncATLAS webserver.

Supplemental Figure S15

A

Overexpression from plasmids (HeLa)

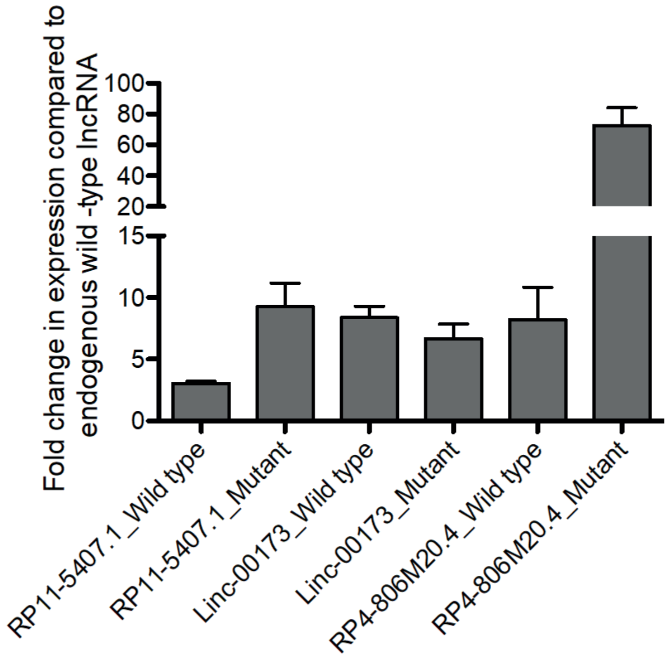

B

Overexpression from plasmids (A549)

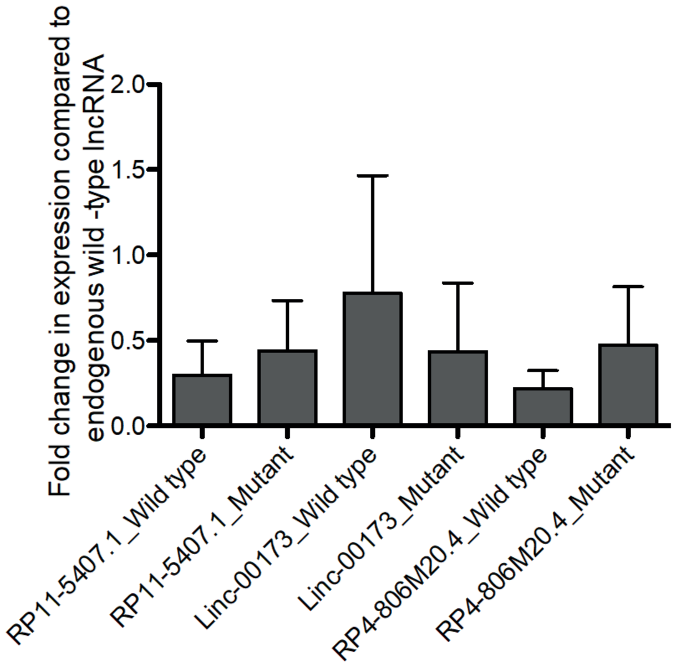

Supplemental Figure S15: Estimating overexpression of transfected lncRNA candidates (wild-type and mutant). Data are shown for the mean of four biological replicates. *y* axis represents the fold change of transfected gene level (normalised to GAPDH) compared to the endogeneous level (normalised to GAPDH) measured from untransfected cells. (A) HeLa cells, (B) A549 cells.

Supplemental Figure S16

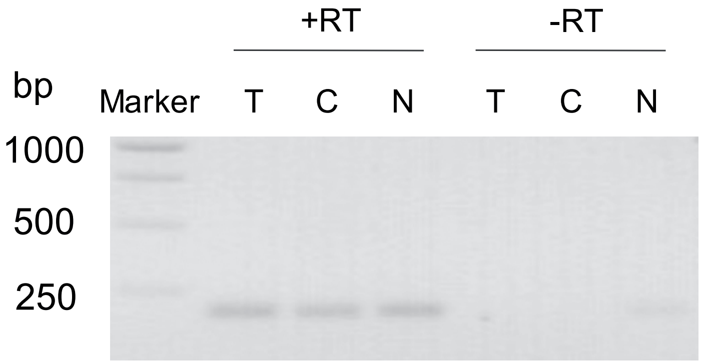

Supplemental Figure S16: DNA contamination check in RNA Isolated from different subcellular fractions. Total RNA was isolated from different subcellular fractions using RNA isolation kit as described in Materials and Methods. Equal amount of RNAs with (+RT) and without (-RT) reverse transcription, were subjected to PCR (40 cycles) with an exonic primer. The reaction products were electrophoresed on a 1.5 % agarose gel stained with SYBR safe. The T, C, N represent the RNA isolated from total, cytoplasmic and nuclear fractions respectively.

Supplemental Figure S17

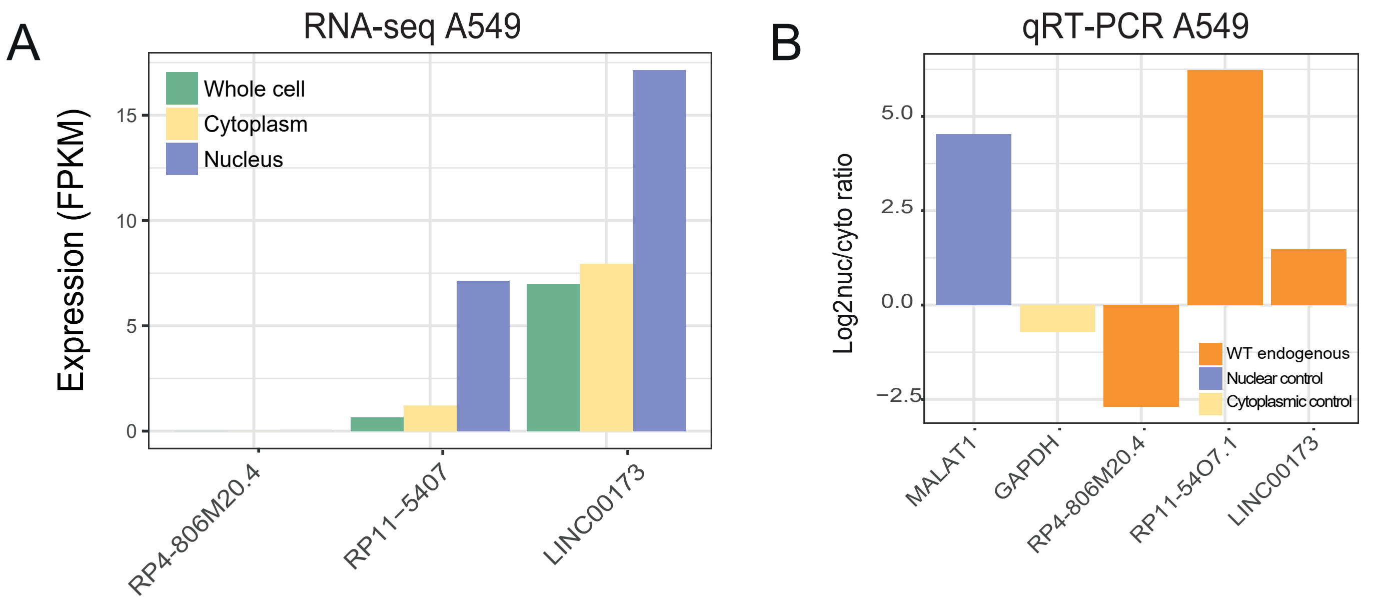

Supplemental Figure S17: Additional candidate lncRNA data for A549 cells. (A) Expression data for three candidate lncRNAs calculated using RNA-seq data. (B) Validation of nuclear/cytoplasmic localisation of candidate genes in wild-type cells by qRT-PCR.

Supplemental Figure S18

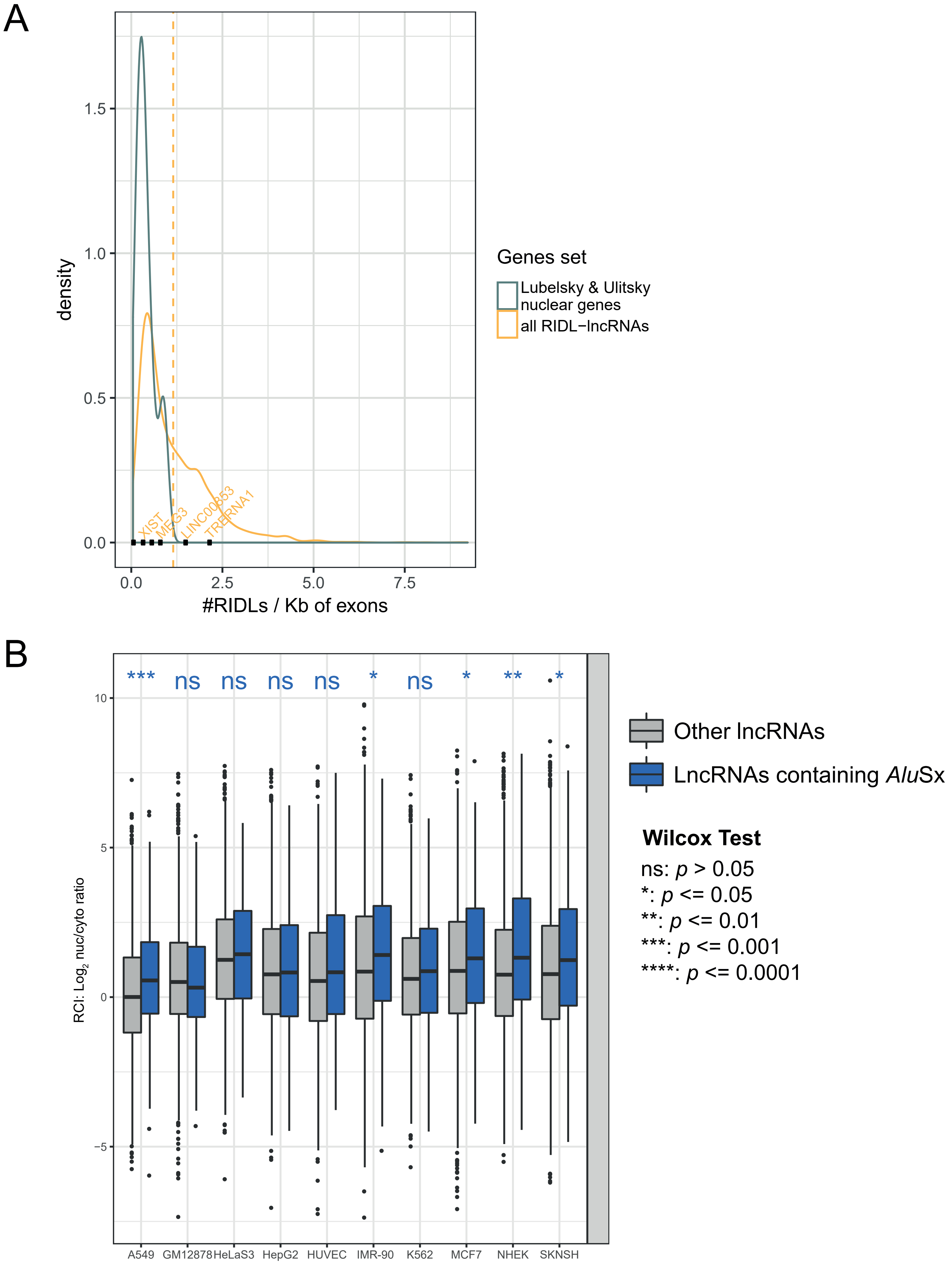

Supplemental Figure S18: Additional candidate lncRNA data for A549 cells. (A) Expression data for three candidate lncRNAs calculated using RNA-seq data. (B)Validation of nuclear/cytoplasmic localisation of candidate genes in wild-type cells by qRT-PCR.

Supplemental Figure S19

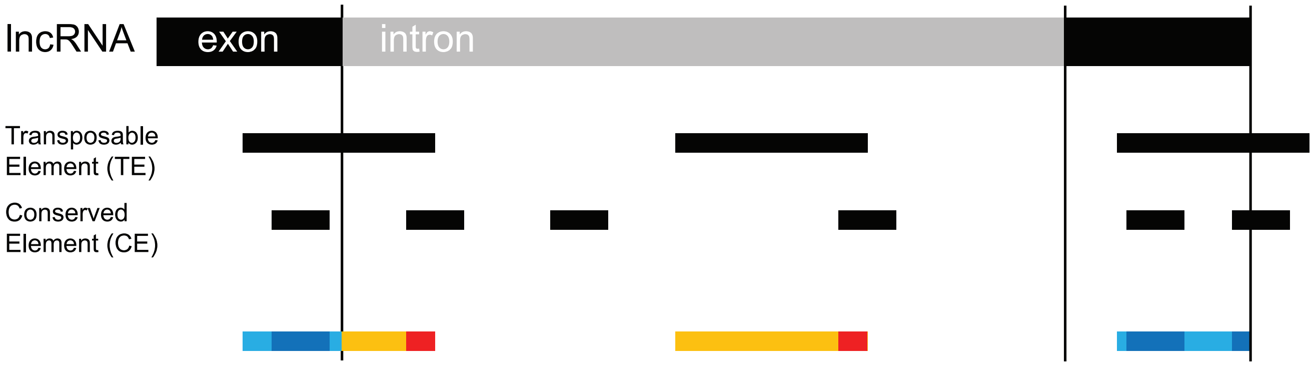

$$\text{Relative Exon Intron Conservation (REIC)} = \frac{\left(\dfrac{\text{Conserved exonic TE nts}}{\text{Conserved exonic TE nts} + \text{Non-conserved exonic TE nts}}\right)}{\left(\dfrac{\text{Conserved intronic TE nts}}{\text{Conserved intronic TE nts} + \text{Non-conserved intronic TE nts}}\right)}$$

Supplemental Figure S19: Overview of evolutionary analysis method
