# Supplementary figures and images for: Ancient exapted transposable elements promote nuclear enrichment of human long noncoding RNAs
Source: Genome Res. 2019 Feb;29(2):208–22. doi: 10.1101/gr.229922.117 (PMC6360812; doi:10.1101/gr.229922.117)

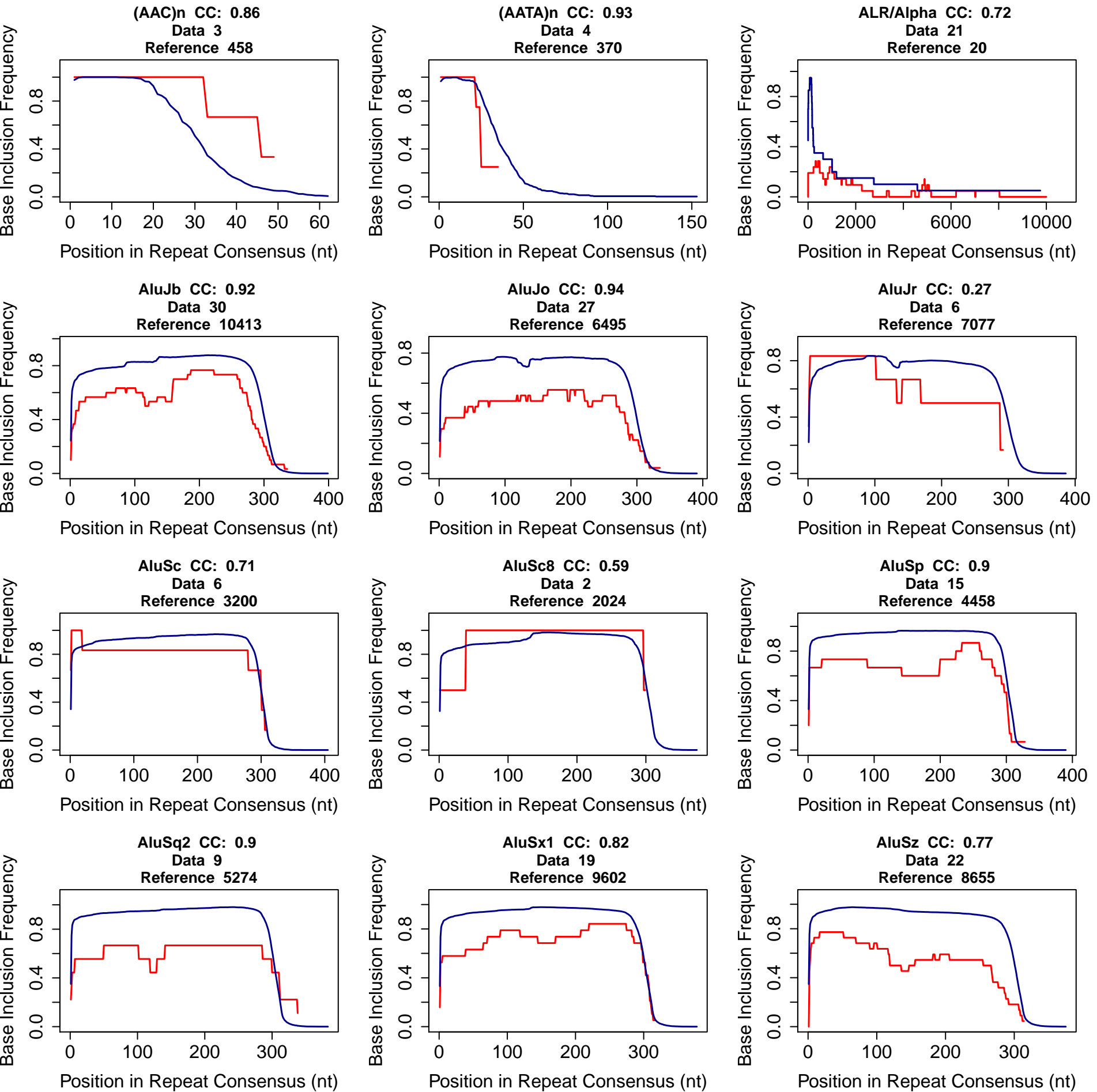

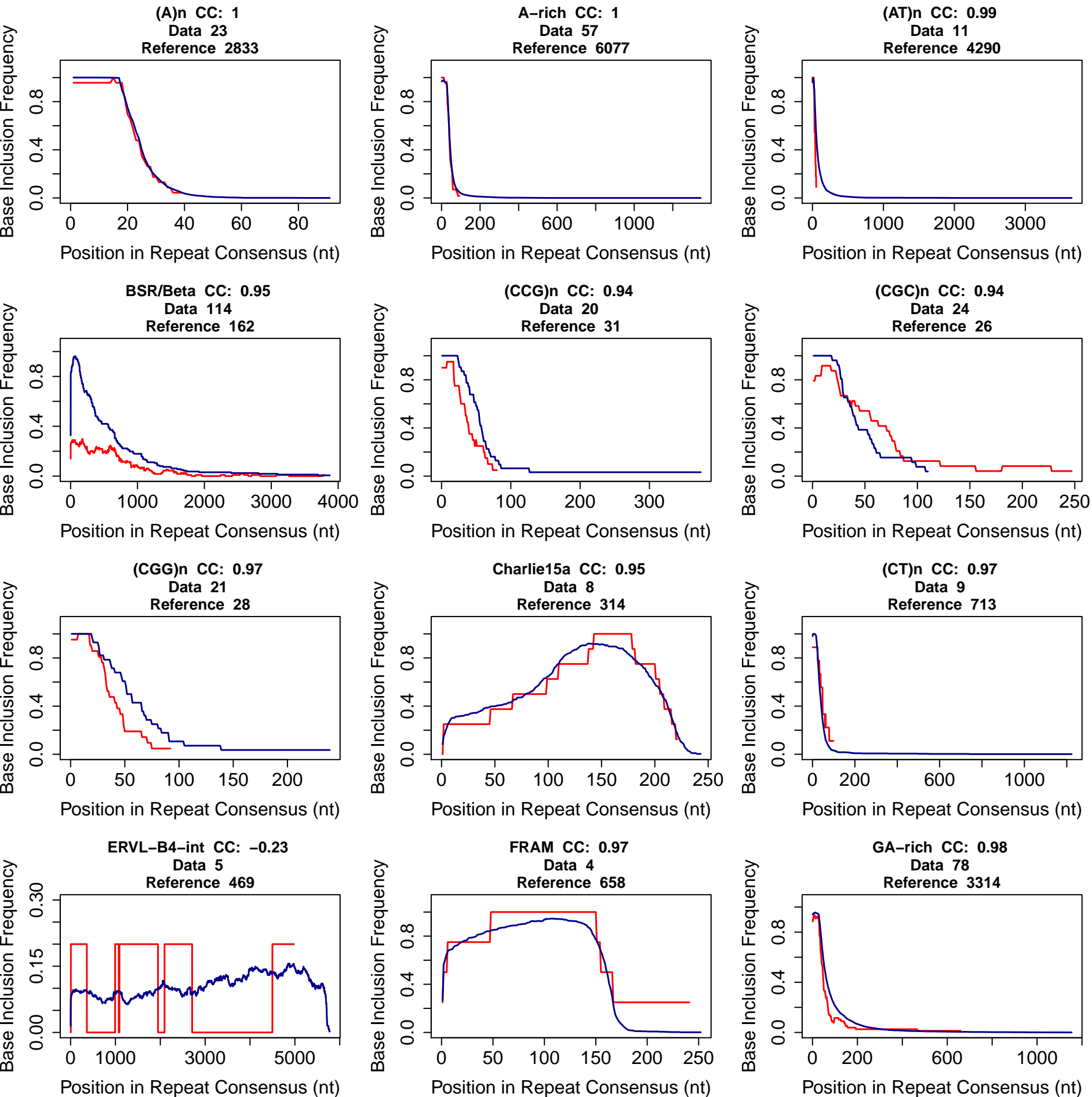

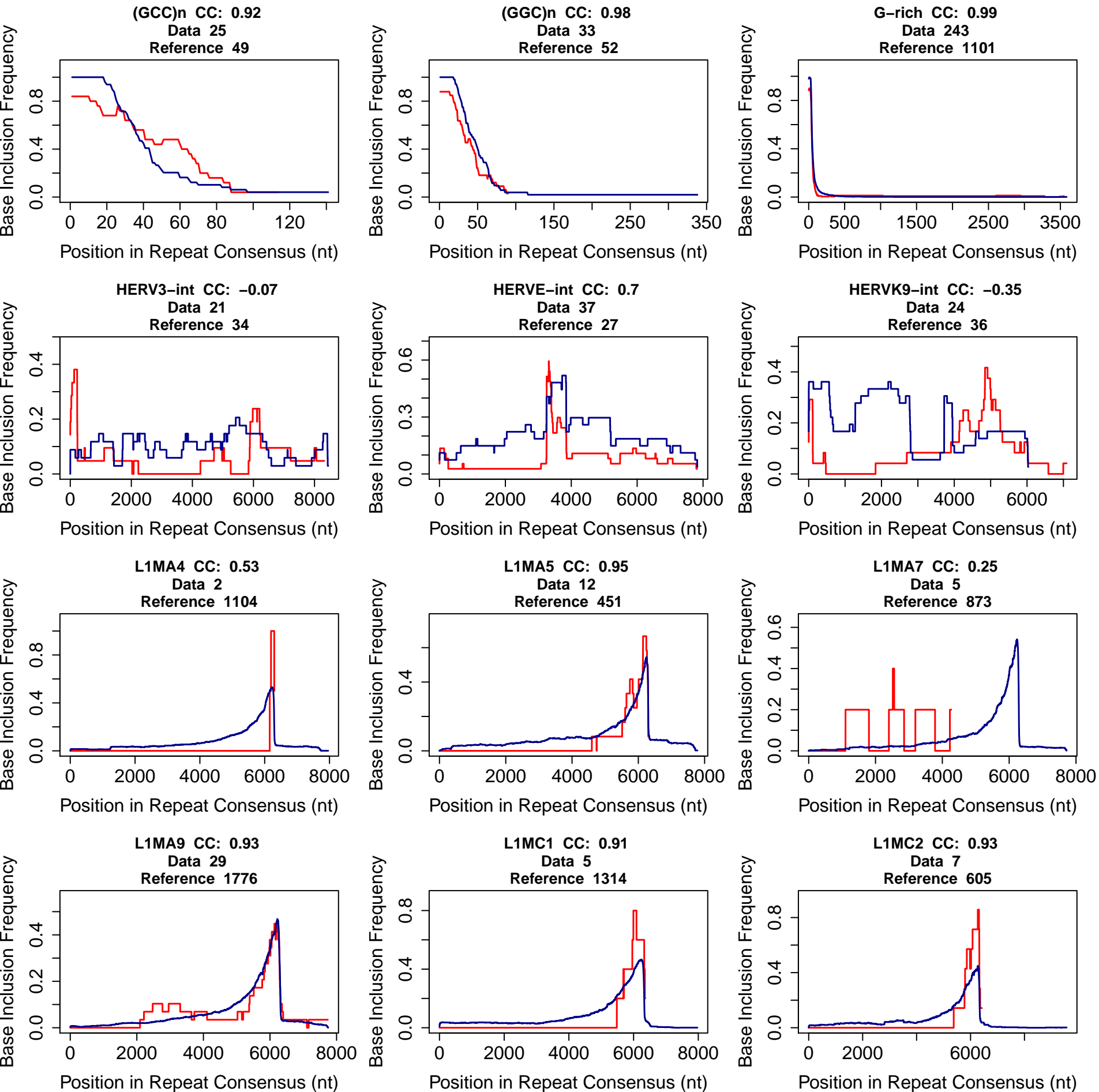

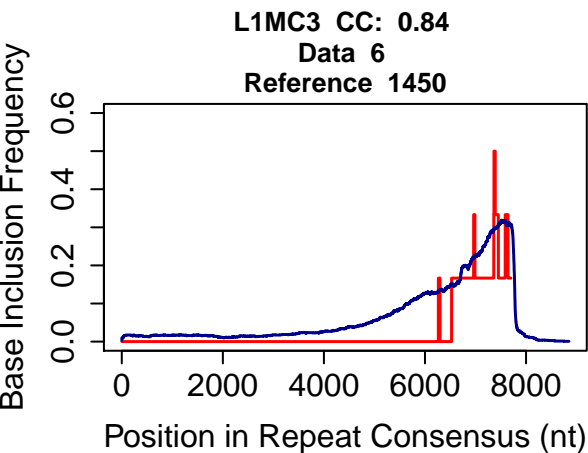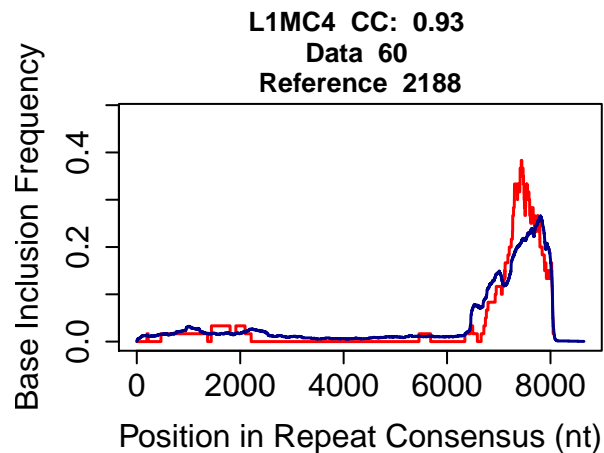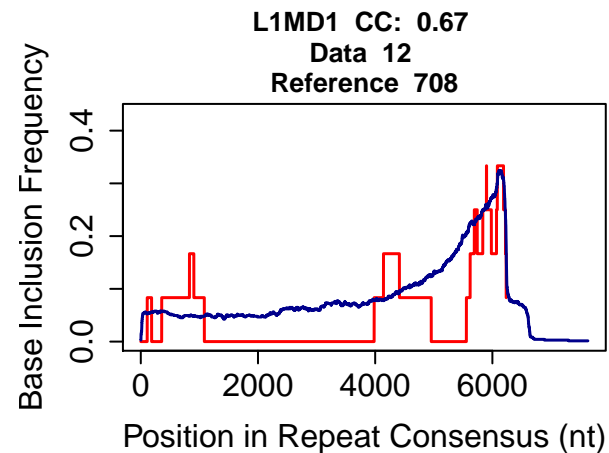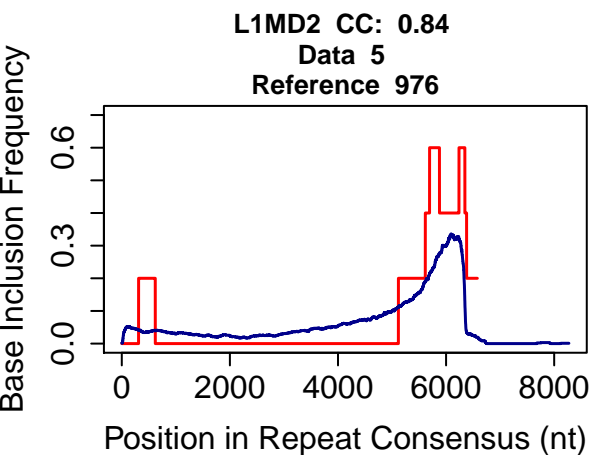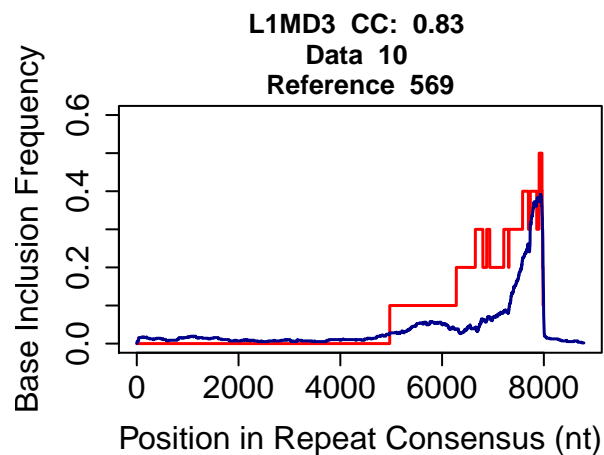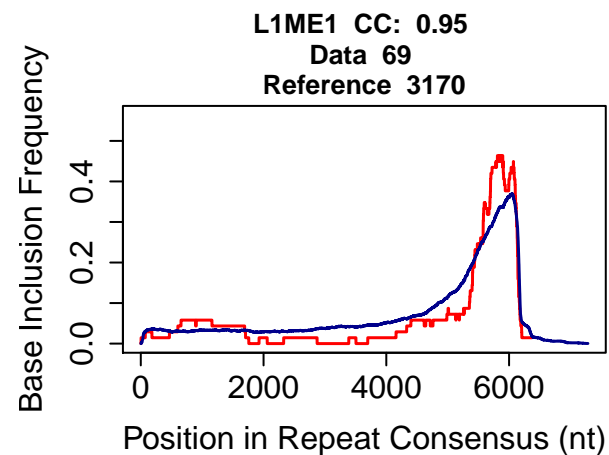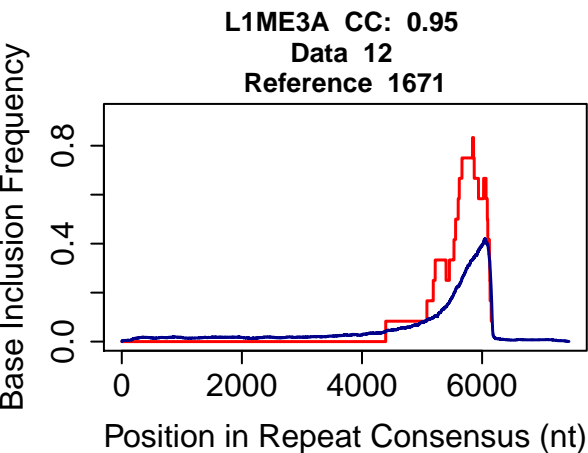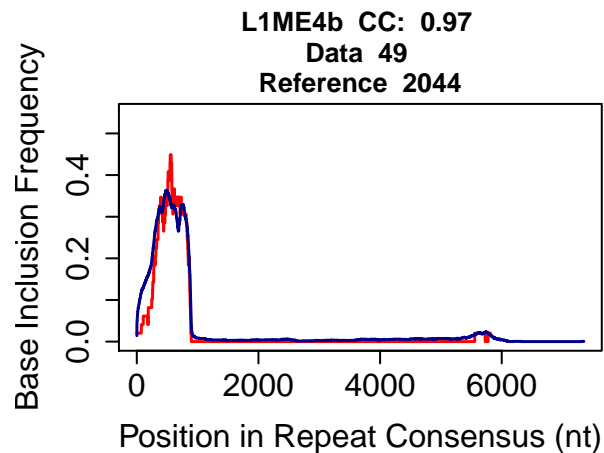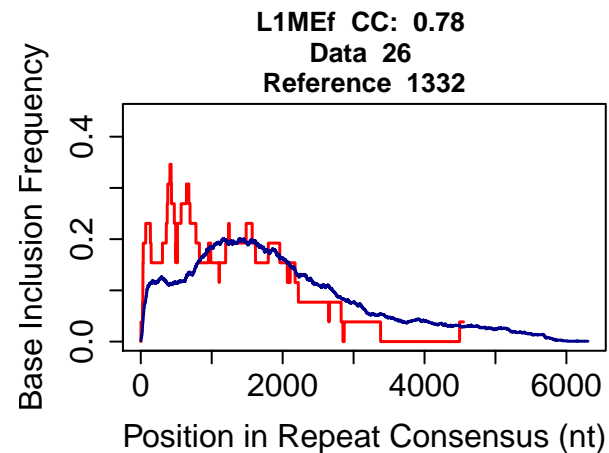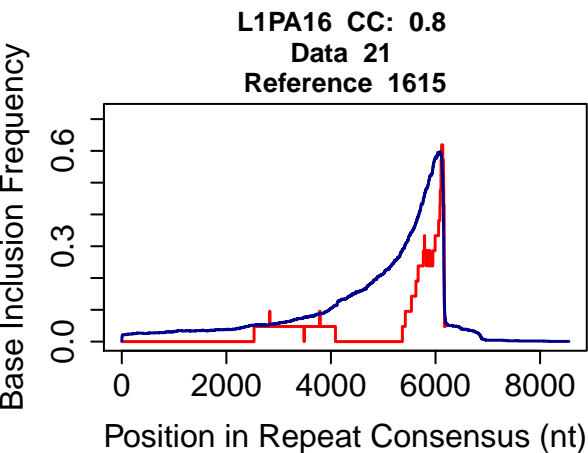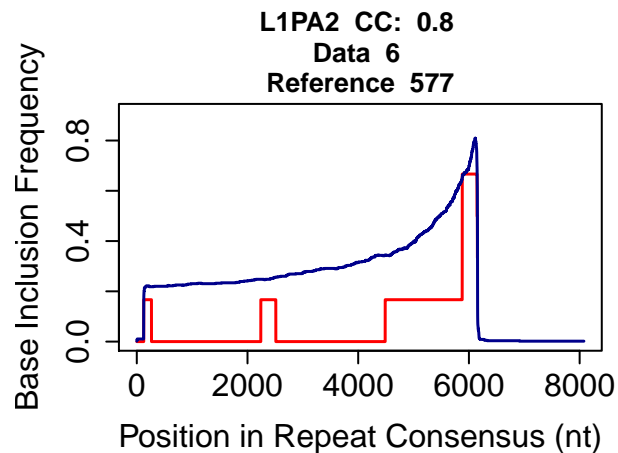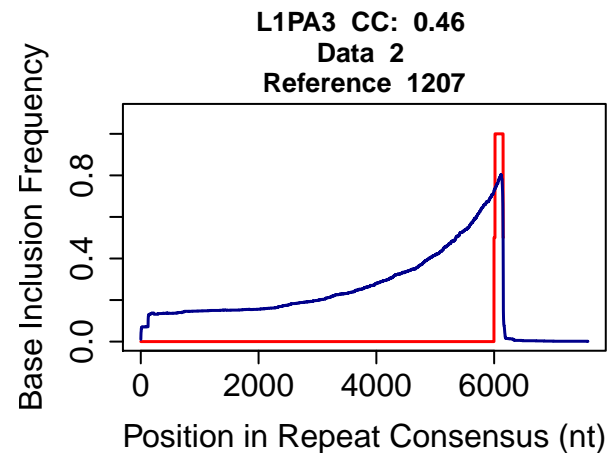



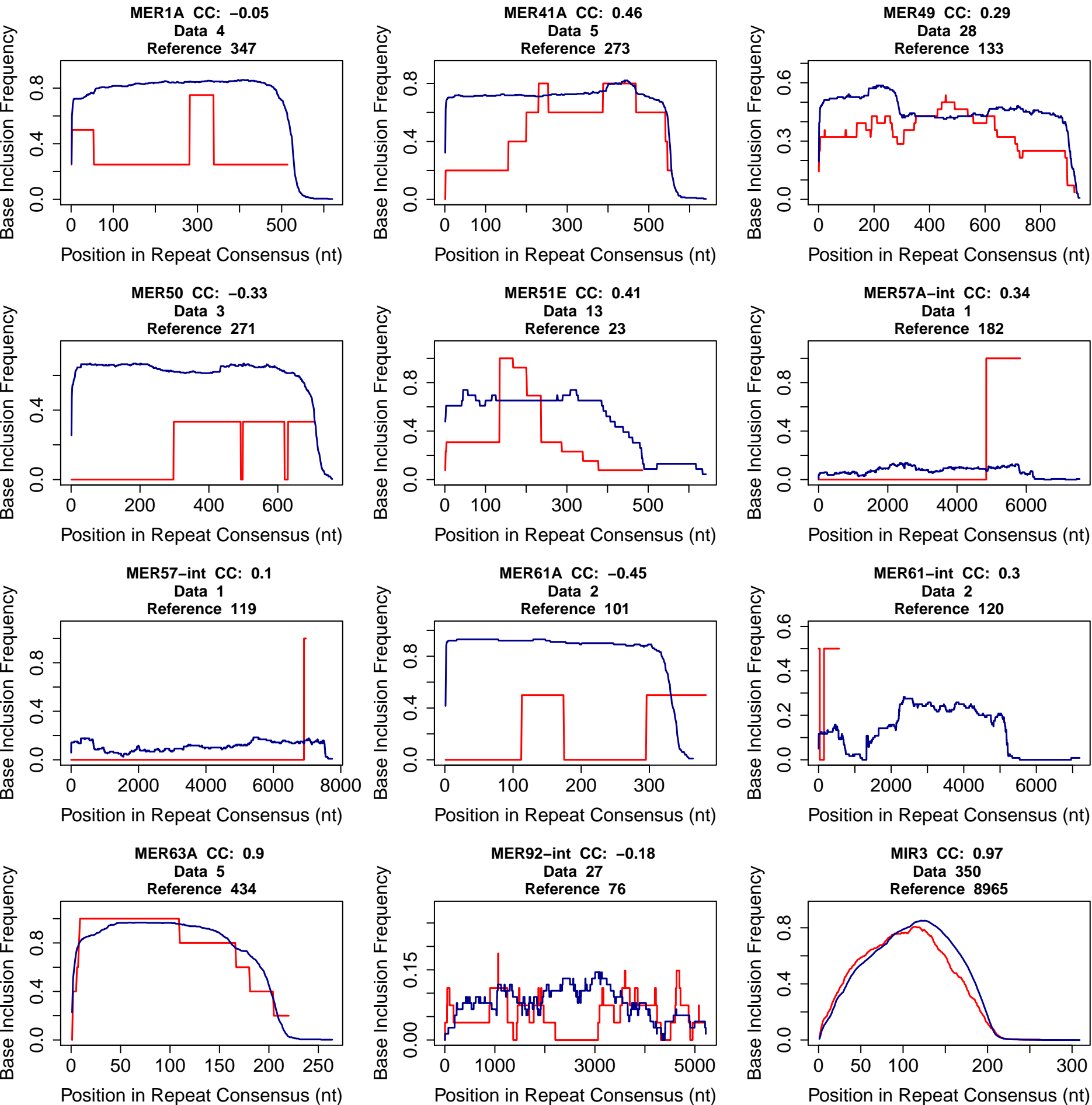

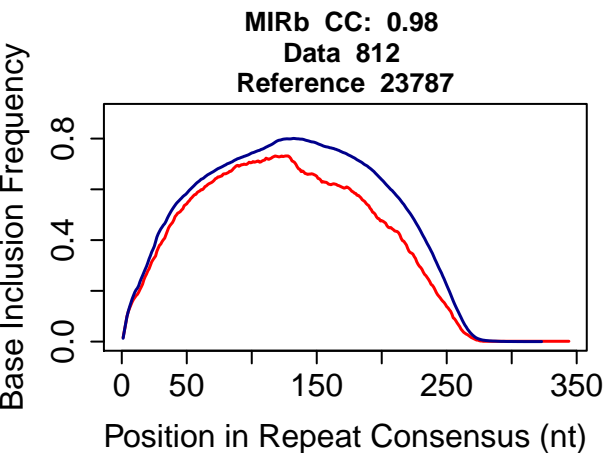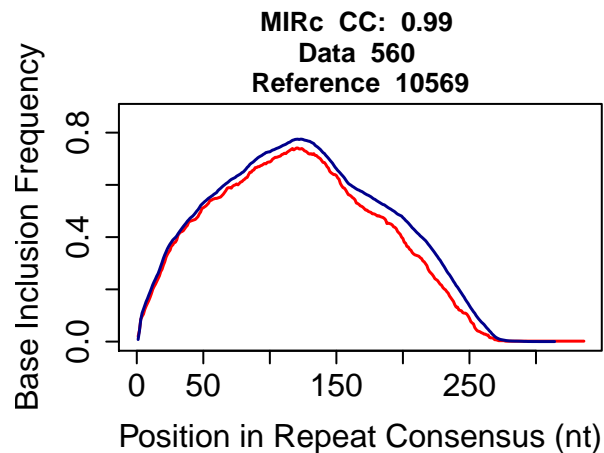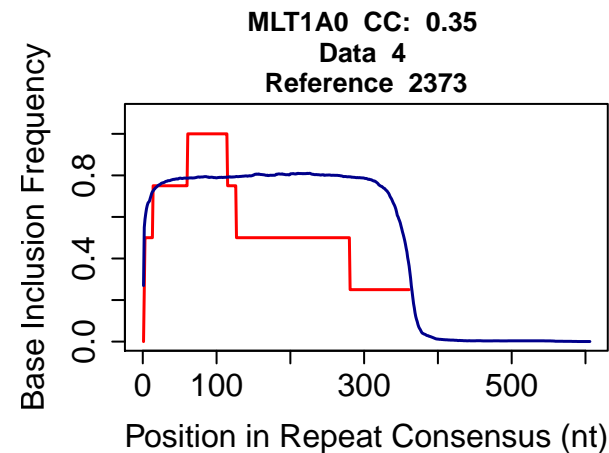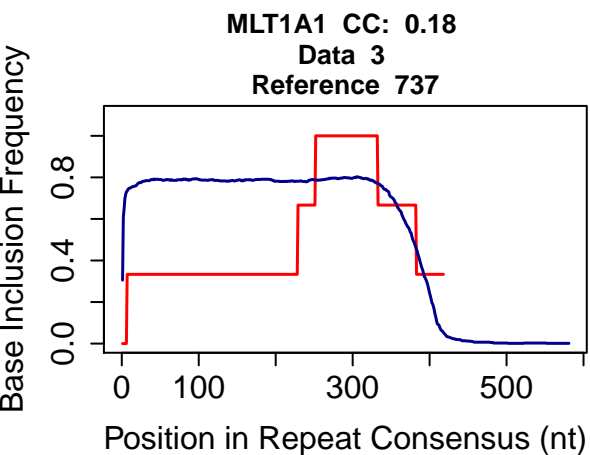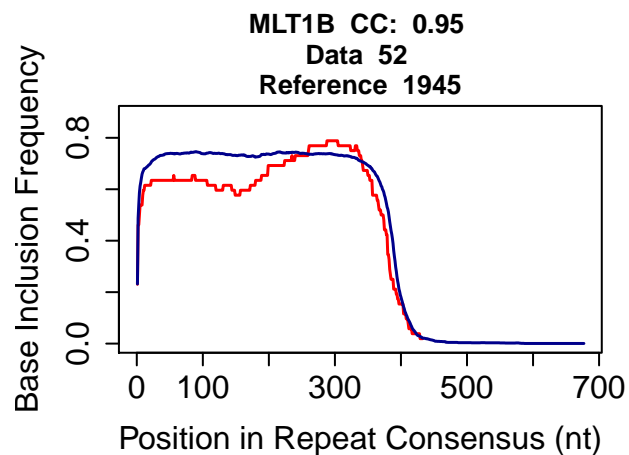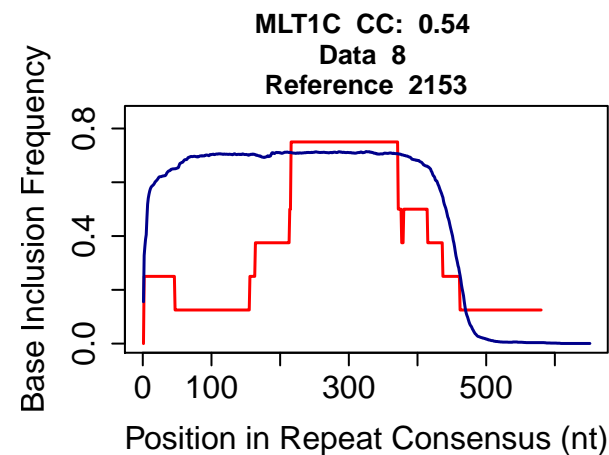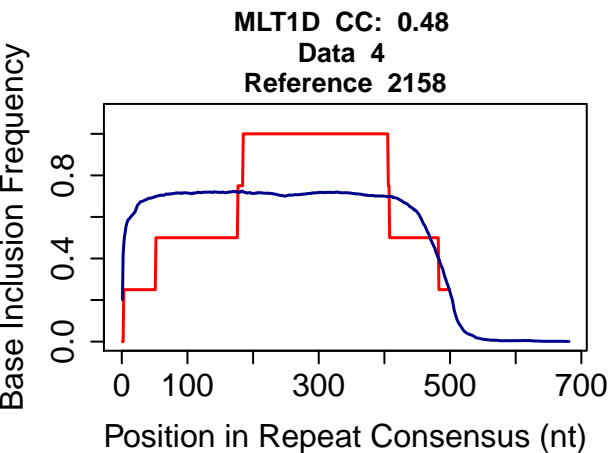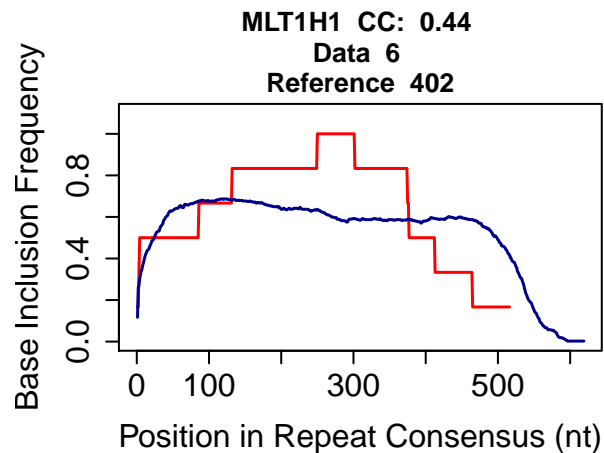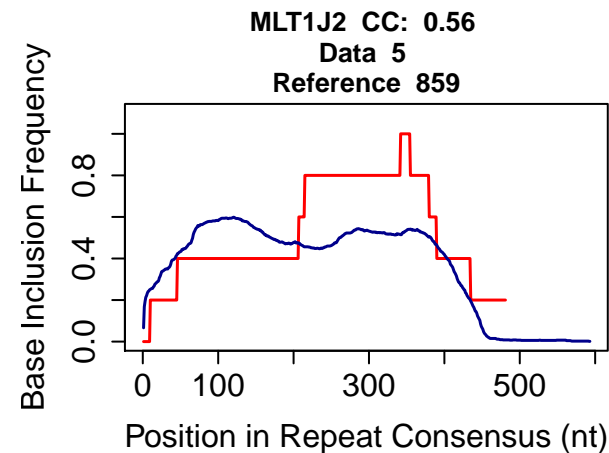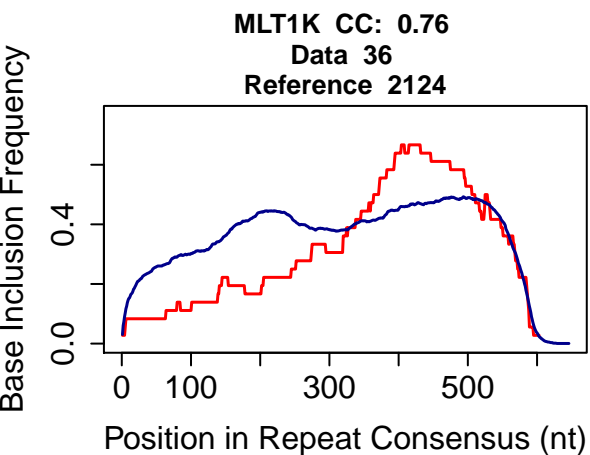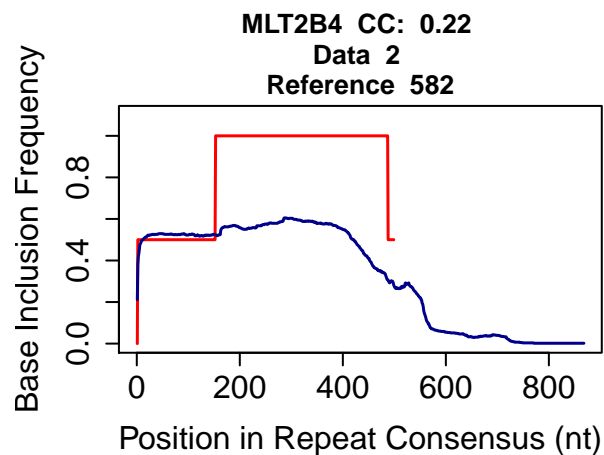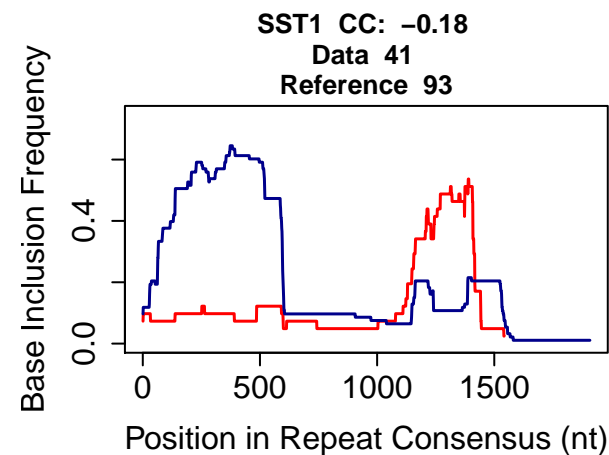

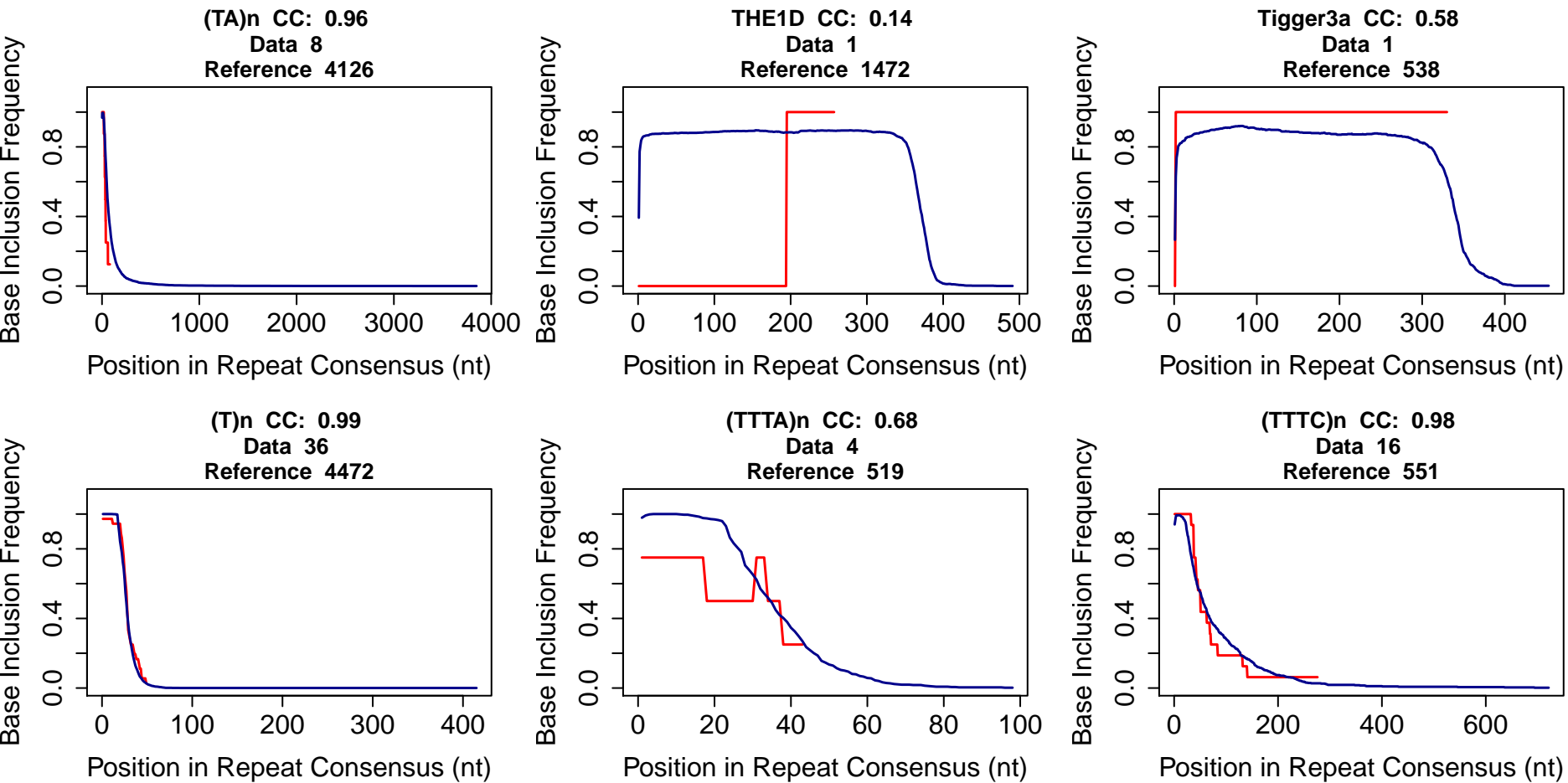

Supplement: Supplemental Material [file supp_gr.229922.117_Supplemental_File_S2.pdf]
