# Supplementary material for: Ancient exapted transposable elements promote nuclear enrichment of human long noncoding RNAs
Source: Genome Res. 2019 Feb;29(2):208–22. doi: 10.1101/gr.229922.117 (PMC6360812; doi:10.1101/gr.229922.117)
Supplement: Supplemental Material [file supp_gr.229922.117_Supplemental_File_S4.docx]

>**RP11-5407_WildType**

GTGCTCACACGCAGCTGGCAATGGCAAGTCAGTGGATTTAGGATAAAACC

GAGAATAAGGAACTTTACATTGTCAATGATCAAACACCTAGTGCACCCAT

GGCCGTGCGTCCGCAGGACAAAGCAGCCTTTGGAGCCCTGGAACTTAAGA

TGTCCAGAGATCAGCAGCTCTCGGCTCAGGCCCAGGCAGCTGGACAGCAC

CCAGCGCCTCCTCCAGGTCCTCCTGGGTCCCCTCCTTTCTTTCACTTTCA

GCCCGGGAATCTCTGTGAAGTCCTAGCTGATAACTAAGGGGTTAATGAAT

AATCACAGTGATGAGCTCTGGAGGAGCCATGGCCCAGAGCAGCTGTGGCC

TCCTTTGATTAAACCACAGAAGTCCGGAGGATCAGGCCTCAAACCTCCAC

CACCTGCCAGCTGGCA**C**CTGTGAAGGAGGTGGGACCAGCACCCACCTCAC

AGTGGCTGCAGGGTCACCTGAGATGGTGATTGCAAAGTGCTGAGCCATCG

GGACACATATTTCCAAGACCTCCTGAGTGCCGGCTGGCCCCTGGAGCAGA

GATGAGACCCCTGTGGGGCCTACAGTCCAGTTATGAAGGCAGGGTTCCTG

TGTAAGCCAGCAGGCCCAGTTCTGGAAAGGCCAGGATGTGGGTTCAGAAG

CTCAAGGACAATGGACTCATTCTGACTGGAGGGATCCAGGAAGGCTTCGT

GGAGGAAGTGGCATTTGACATTGGCATCAAGGATGAACAGGAATAGAGAG

AGGGGACTGGTGTCTGCGTGAAGGATCGCCAAAGCGCTGTTCTGGGGTGA

GGGCGATCAGCCCCTCCTGCCCCTCCTGAACTATGAGGCTCGGGAGCCTT

TGGCAGAAGGTGGCTCTGCACGCCCCAGGTGCAAGCTTGGAGCTTCCTTT

TCTGCACTCCACAGCAGGAAGAAGGGCAGTGTGAACTGCTCACCACTGTC

TTCCTGGGTTTGCAGCTCTGCGCCTGGCACAGAGACAGGCTCAGTGACGG

TTGAAGGAATCGGAGAAATAATGAAAAGTGTGTTCATCCTGCAGGCAGCA

GAGCCTCGGGTTAACCGTTAACGTCAGGAAATGACAGATCCAACGCTCCT

GCTTGGGGCAGCGTGGAGGCCAGCGGAGGAACTGCAGGAGCCTAAAGGAG

ATGGCGCGCGGGCCTGGCTCAGGAGAGCAGCCGTCTGAAAGATGGCGGCG

AAGGGTGCGGGAGGAGATGCCCCGGGCAGGGGCTTGGCGGGGTCTGGCAC

CAAACTTCCCAAAGCAGCTGCCTCTGTGAACTGTGCCTGGCAGGGAGGGA

GCCACACCGGGAGTGACCCTAGACGCTGGCTCCCCAGGGGTGACTGGAGG

CAGTGTCTCCCGCCCATGTGGGCCTCCGGCTTGGGGTCCCCAGAGCTCTG

CCCTGCTGCTGGCTGCACTGAGAGAGGTGGCCTTCTGAATGGGCCCTGTC

CCCGGGGAGCCTCTGGTAAAGAGATTCACCTGCACCCTCAAAACACGCCA

TGCCCATTGGAGGCTCTGGGTGCAGGCTGCCCCTCAAACAGAGAAAAACC

ACACGCTGTTTAAGACCCAAACCAAGGAGACAGCCCCTCATGCCCCCTTC

TATCTCCCCTTCTGTCCAGGGCCCCCTGCCTGCTCTGGTCCACCCCGTCC

TGACCCCCTCTGCCTGTTCATCTGAGGGCCGTGAGCCAAGGCCTGGGCCT

GGCCAAGGGTCAGGGATCCTGGGACCTGCTTGAGACCATGCCCCATACAC

TCCTTCGGCCAGAAGGTCAGCCGTAGAGACAAACCCCCACCCTGAGCCCG

CAGTGTCAGCAACGCCCTGTCCTCAGTGCCAGGCAGTGTCAGGCTGAGAC

CCGGGCCACCTGATCCAACGTCCACTCCAAGCAAGCCAGAGCCAGGTGCT

GCCAGCCTGCCCATGTGCACACGTTAGCAGACAGGTTTCCTGGTGTGGCA

CACACGTGTTATGACCGCGGCTGCTGCCCCCAGCCCAGAGTGCACTTCCG

GGTAATCCAGCACCCTGAGGCTTCACTGCTCTCCTGAGAGTTTTCTAAGA

TTAAATCTCCTTCACAATGACAGCCTCCTGTCACCTGGGCCCTGGTGACA

AAGCCTTTGGAGAGGCAAGGACCCAGCCGGGGCATTCACAGTGGGCATGC

TATCCACCCTCCGCCCCTGTCCTGGCCCCCACAGTGGAGCCGCCACCTTG

GGTAACAGGGGCTGCCTGAGGTCCTGGAGAAGGAAGAACCAGGTGGCCCA

CCTTCGGTGCACAGGGAACAGCAGGGACCTGACGGCAGCCCCCGCACCCC

TCCCTGGGCTGAAGTTCAGGCTGTCTCATCCCTTGCCAGGAGCTCAGGGC

TGAGCTAGGTTGGCAGGGGATGGGTTGAGGGTCTGTTGTCCAGCAGATTG

CCCGGGCCCCACCCAGGCCACTCAGAAAACACCTGTGAGAGCCCCGCAGG

CATGCAGGGCACTGTCGTGAGCACACCAGTGGGGACAGACGTCCAGGCAG

GAGGGAGCAGCCAGACCCCCATCCCGCCAGGGTCACCAGGTGAGCAAGGC

TGGGGAACAATGGAGGCGGAGCCCCGCGACTGCCCAGCTCACTGCCCCCA

GTGTCCAGGCTGTGGTGCAGGCGTGGGGACCCAGGTGTGTTCAGGGGTCT

CCTTCAGGGAAGGAGACCAAGCTGGGAGTCTGGGGAGGCTGAGGTGTGAG

GTGACAGCAGGTAAAGAGTGAGCCACAATCAGCATTTTCACATGAAAGGA

AACTACAGGCCTGGGCGCAGTGGCTCACGCCTGTAATCCCGGCACTGTGG

GAGGCTGAGGTGGGAGGATCACCTGAGGTCGGGAGTTCGAGACCAGCCTG

ACCAACATGGAGAAACCCCATTTCTACTAAAAAAAATACAAAATTAGCTG

GGCATGGTGGCGCATGCCTGTAATCCTAGCTATTCGGGAGGCTGAGACAG

GAGAATCACTTGAACCCGGGAGGCGGAGGTTATGGAGAGCCAAGATCGCA

CCACTGCACCCCAGCCTGGGCAACAAGAGTAAAACTCCGTCTC

>**RP11-5407_Mutant**

GTGCTCACACGCAGCTGGCAATGGCAAGTCAGTGGATTTAGGATAAAACC

GAGAATAAGGAACTTTACATTGTCAATGATCAAACACCTAGTGCACCCAT

GGCCGTGCGTCCGCAGGACAAAGCAGCCTTTGGAGCCCTGGAACTTAAGA

TGTCCAGAGATCAGCAGCTCTCGGCTCAGGCCCAGGCAGCTGGACAGCAC

CCAGCGCCTCCTCCAGGTCCTCCTGGGTCCCCTCCTTTCTTTCACTTTCA

GCCCGGGAATCTCTGTGAAGTCCTAGCTGATAACTAAGGGGTTAATGAAT

AATCACAGTGATGAGCTCTGGAGGAGCCATGGCCCAGAGCAGCTGTGGCC

TCCTTTGATTAAACCACAGAAGTCCGGAGGATCAGGCCTCAAACCTCCAC

CACCTGCCAGCTGGCTGTGAGGAAAGTCCGTCATTAGGCGAACCCCGCTC

ATACCCGCCGATGAGATGAGGGGCCGGCACGGTGGATCTGCTGAACATCG

GGACACATATTTCCAAGACCTCCTGAGTGCCGGCTGGCCCCTGGAGCAGA

GATGAGAGCTGATCGAACTACCTTACCTGGGTTCTTCCCTACCACGTTAC

CCGAGCCTCTCCAATCGAGACATCACGGCACATTGAAATGGTTCATCACT

ACCCCATGTCAGATCACCAAGCGTCTGCCTGGTCACTGGGCTGTAAGCTT

TCTGCCTGACCCTTCCAGGTCGTATCCGATGCTATCTACGCGAGAGAGAG

AGGGGACTGGTGTCTGCGTGAAGGATCGCCAAAGCGCTGTTCTGGGGTGA

GGGCGATCAGCCCCTCCTGCCCCTCCTGAACTATGAGGCTCGGGAGCCTT

TGGCAGAAGGTGGCTCTGCACGCCCCAGGTGCAAGCTTGGAGCTTCCTTT

TCTGCACTCCACAGCAGGAAGAAGGGCAGTGTGAACGCAGCCCTCTGTAC

GGTTGGTAGCTTTATGACCTCTTGAGCTCCTCTCAGCCGAAGTAAGGAGC

TCACGGGGGACGGAGAAATAATGAAAAGTGTGTTCATCCTGCAGGCAGCA

GAGCCTCGGGTTAACCGTTAACGTCAGGAAATGACAGATCCAACGCTCCT

GCTTGGGGCAGCGTGGAGGCCAGCGGAGGAACTGCAGGAGCCTAAAGGAG

ATGGCGCGCGGGCCTGGCTCAGGAGAGCAGCCGTCTGAAAGATGGCGGCG

AAGGGTGCGGGAGGAGATGCCCCGGGCAGGGGCTTGGCGGGGTCTGGCAC

CAAACTTCCCAAAGCAGCTGCCTCTGTGAACTGTGCCTGGCAGGGAGGGA

GCCACACCGGGAGTGACCCTAGACGCTGGCTCCCCAGGGGTGACTGGAGG

CAGTGTCTCCCGCCCATGTGGGCCTCCGGCTTGGGGTCCCCAGAGCTCTG

CCCTGCTGCTGGCTGCACTGAGAGAGGTGGCCTTCTGAATGGGCCCTGTC

CCCGGGGAGCCTCTGGTAAAGAGATTCACCTGCACCCTCAAAACACGCCA

TGCCCATTGGAGGCTCTGGGTGCAGGCTGCCCCTCAAACAGAGAAAAACC

ACACGCTGTTTAAGACCCAAACCAAGGAGACAGCCCCTCATGCCCCCTTC

TATCTCCCCTTCTGTCCAGGGCCCCCTGCCTGCTCTGGTCCACCCCGTCC

TGACCCCCTCTGCCTGTTCATCTGAGGGCCGTGAGCCAAGGCCTGGGCCT

GGCCAAGGGTCAGGGATCCTGGGACCTGCTTGAGACCATGCCCCATACAC

TCCTTCGGCCAGAAGGTCAGCCGTAGAGACAAACCCCCACCCTGAGCCCG

CAGTGTCAGCAACGCCCTGTCCTCAGTGCCAGGCAGTGTCAGGCTGAGAC

CCGGGCCACCTGATCCAACGTCCACTCCAAGCAAGCCAGAGCCAGGTGCT

GCCAGCCTGCCCATGTGCACACGTTAGCAGACAGGTTTCCTGGTGTGGCA

CACACGTGTTATGACCGCGGCTGCTGCCCCCAGCCCAGAGTGCACTTCCG

GGTAATCCAGCACCCTGAGGCTTCACTGCTCTCCTGAGAGTTTTCTAAGA

TTAAATCTCCTTCACAATGACAGCCTCCTGTCACCTGGGCCCTGGTGACA

AAGCCTTTGGAGAGGCAAGGACCCAGCCGGGGCATTCACAGTGGGCATGC

TATCCACCCTCCGCCCCTGTCCTGGCCCCCACAGTGGAGCCGCCACCTTG

GGTAACAGGGGCTGCCTGAGGTCCTGGAGAAGGAAGAACCAGGTGGCCCA

CCTTCGGTGCACAGGGAACAGCAGGGACCTGACGGCAGCCCCCGCACCCC

TCCCTGGGCTGAAGTTCAGGCTGTCTCATCCCTTGCCAGGAGCTCAGGGC

TGAGCTAGGTTGGCAGGGGATGGGTTGAGGGTCTGTTGTCCAGCAGATTG

CCCGGGCCCCACCCAGGCCACTCAGAAAACACCTGTGAGAGCCCCGCAGG

CATGCAGGGCACTGTCGTGAGCACACCAGTGGGGACAGACGTCCAGGCAG

GAGGGAGCAGCCAGACCCCCATCCCGCCAGGGTCACCAGGTGAGCAAGGC

TGGGGAACAATGGAGGCGGAGCCCCGCGACTGCCCAGCTCACTGCCCCCA

GTGTCCAGGCTGTGGTGCAGGCGTGGGGACCCAGGTGTGTTCAGGGGTCT

CCTTCAGGGAAGGAGACCAAGCTGGGAGTCTGGGGAGGCTGAGGTGTGAG

GTGACAGCAGGTAAAGAGTGAGCCACAATCAGCATTTTCACATGAAAGGA

AACTACAGGCCTGGGCGCAGTGGCTCACGCCTGTAATCCCGGCACTGTGG

GAGGCTGAGGTGGGAGGATCACCTGAGGTCGGGAGTTCGAGACCAGCCTG

ACCAACATGGAGAAACCCCATTTCTACTAAAAAAAATACAAAATTAGCTG

GGCATGGTGGCGCATGCCTGTAATCCTAGCTATTCGGGAGGCTGAGACAG

GAGAATCACTTGAACCCGGGAGGCGGAGGTTATGGAGAGCCAAGATCGCA

CCACTGCACCCCAGCCTGGGCAACAAGAGTAAAACTCCGTCTC

**>RP4-806M20.4_WildType**

AGGCCCAGCCTCATGTGGGAGGTGAGGAGAAAGGACCCTTCCAGAGGGCC

CCCCCTCAATCCTGTTGTGCCTAATTCAGAGGGTTGGGTGGAGGCTCTCC

TGAAGGGCTCTGAAGAGCGCTGCACCCGCAGCTGTGTGCCAGGAGTGGAG

ACAGGACAGTCGATACAAGAGGGGCCCCTGTCATGTCAGCTGCTGAGTCC

CCTGGGGCGGCCGGGCACTGGGGAATGGTGAACTCGCCGTGCCGCGCCTC

TGCCACAGTCTCCCGTGGGTCCCCACTGCCGGGAGTTTTACCTACCTCCC

ACCCTCAGCCACGAGCACCTGCAGGATGCGGTGCCATCCTCGCCGGTGCC

CGCTCCCCTTCCGGAGGCTCTCTCCACCCTCACTGACGCCTCCTCGGGAA

AGCCCCCGCTCCTTCCCCGACCTCACAGCATGCCAGGTCTGGGGCTTCCG

CTGGGGGGCTTACCTGTTTTCCACCCCTGCTAGACTGGGAGCTCCAGGGC

AAGGCCGTAGCTGCCTTGCGGATTGTTACGTTGTCCAGCTTGTCTCCACA

CGTGATACCCAGCAGGTGCCTAATAAACTTGTGTAGAAAGAGTG

**>RP4-806M20.4_Mutant**

AGGCCCAGCCTCATGTGGGAGGTGAGGAGAAAGGACCCTTCCAGAGGGCC

CCCCCTCAATCCTGTTGTGCCTAATTCAGAGGGTTGGGTGGAGGCTCTCC

TGAAGGGCTCTGAAGAGCGCTGCACCCGCAGCTGTGTGCCAGGAGTGGAG

ACAGGACAGTCGATACAAGAGGGGCCCCTGTCATGTCAGCTGCTGAGTCC

CCTGGGGCGGCCGGGCACTGGGGAATGGTGAACTCGCCGTGCCGCGCCTC

TGCCACAGTCTCCCGTGGGTCCCCACTGCCGGGAGTTTTACCTACCTCCC

ACCCTCAGCCACGAGCACCTGCAGGATGCGGTGCCATCCTCGCCGGTGCC

CGCTCCCCTTCCGGAGGCTCTCTCCAGTCGCGGATCTTCATATTGGGCGA

ACACGTACTCCGAGGACGCTTATGTTCTGCCGATCCTACATTCGGGCCTC

CCTTCTCTCCAGCTGAGTGGCCTAATTGTCTGTGTAGGGCGTGGATGGCA

CCGCGCAAGTCCCCCTAAGGAGACGACGCGTCTCTCCCTACCCCCGCCGC

TACCACGGCTGGCCGCAGCGGTTCTTGATACCACAGTAGAACCT

**>LINC00173_WildType**

AGCCTTCTGGGTCCGAGGCTCCCACCTGCTCTAAGCGCTTGACACCCTTT

AAAAAAATGTATTTAAAGAGGCTGGTTCCTATCCATCCGACTGGAGGCAT

CTCAGTGCAAGAGCAAAGCTAAGTCCTGCACACGCTCCTCCCCTCCTCCT

CCTCCTTCTCCCCCCAGGTTTTCCCGAATGTATCTACTCCGGTTACAACT

AGACGCGGCCCCTCCCCCACCTGCCTCCCCCCTTCCTTCCCTCGATCGTG

GAGGGAGCGTTCTCTGTGCCTTCCCAAGTCCCCGTGGGGGACCTTCTATG

TTGGAGTGGGGGGAGGGGGGGAGGGTCATATAACGAAGGCCAGAAAGAAC

AAATTAGATAATCAAAAGAATTATAGTAATTGCTTTCACTTTCCCCCGCC

CGCTCAGCGGATTCCCTCCCCCGCCCCTCCCCTGGTTTTTCTGTCTGTCG

GGAATACTCGGTCTTTCCGACCCCCTCCCCTCCCCCAGGTTCCTCCTCTC

CTCTCCCCTTGCTCGCGCGTTCCCTCTCTTCCTCCGTTTTCTGGTGTGCT

GGAACGTTCAGCGGAATATGATGAATGATCACCTGTCACAGCTTGTTTAT

TATAATGCAGGCAATCAATTACACATCCCCAATGCTGGCCGGCCCGCAGG

AAATTTATATGCTCAGCACAAACCAATGTGAAAATGGAATCTCATTTGCC

AAATGTCTTTCTCCCCGTACAGCACGATGATTACAGTCTGTGTTTGTTTC

AACAGTCGTGTACAACTGACAGTGCCATCATTTACTGCCTGGCTCAGGTC

ACGTTACTCTAAGGCTTTATTTATGGTGTTACGAAGGGCAGCACAGGAAA

AGGACAAGGGTGTCTGTCAGGGATGGCACTGTGTTAAAAAGTGGGCGTGC

AAGGGCCGCATTCCCGGGCAGCCGCTGCAACCTCAGCCCCTGGGCCCTTA

CCTCCGCAGCCTCTCCCAGCATCCAGCTACCCAGACTCCAAGGCCCCAGG

CGAGAGCCAGCTCTCGGTACCTGGAGCTCCACAGGTCCCAGAATCGGGGT

GGATCAGAGTTCAAATTCTGGTTCTGCTACTGTCTAATTGCGTGCTGCAG

GGACTCAATCTCTTCATCTGGGAAATGGGAGTAATAACCCTTGGCAGGAA

TGTTGCGATCCTCTGGGATGTCAGAGGTGTTGATGAATGTTAGTTCCCGG

GACTTCGGAAAGAGGTCCCGTTGGAAGAGATGTGAATTGGAATTCACACC

CTATATTAAAATCTCCTCCAATCTTCACCTCTGAGACATGGCTGTCTCAA

GACTGTTTTGTTTCCCTTCCTGGTGGAATTTTGCACTTTTATGTCCTGTG

TAGCAGCAGGTAGTGTGGCTTTGAGAAAATAAAATGGCCACCTTGCTCCG

CTGTTCTTTCTTTGTAAAAAAAAAAAAAAAAAAAAAAAAACGGCATAGCA

ATCTTGGCCTTTCTAGCTGTGTGACCCCAGGCCGGTCAATCCCTCCTCCT

CTCCAAGCCTCGGATTCCTCCCCTGAGAAGTAAAGAAAATAACTCCTAAA

CTGCCTCCCGAGGCTTGCTGGCAGGATCCAAGGTGTCCAGAGATGTT

**>LINC00173_Mutant**

AGCCTTCTGGGTCCGAGGCTCCCACCTGCTCTAAGCGCTTGACACCCTTT

AAAAAAATGTATTTAAAGAGGCTGGTTCCTATCCATCCGACTGGAGGCAT

CTCAGTGCAAGAGCAAAGCTAAGTCCTGCACACGCTCCTCCCCTCCTCCT

CCTCCTTCTCCCCCCAGGTTTTCCCGAATGTATCTACTCCGGTTACAACT

AGACGCGGCCCCTCCCCCACCTGCCTCCCCCCTTCCTTCCCTCGATCGTG

GAGGGAGCGTTCTCTGTGCCTTCCCAAGTCCCCGTGGGGGACCTTCTATG

TTGGAGTGGGGGGAGGGGGGGAGGGTCATATAACGAAGGCCAGAAAGAAC

AAATTAGATAATCAAAAGAATTATAGTAATTGCTTTCACTTTCCCCCGCC

CGCTCAGCGGATTCCCTCCCCCGCCCCTCCCCTGGTTTTTCTGTCTGTCG

GGAATACTCGGTCTTTCCGACCCCCTCCCCTCCCCCAGGTTCCTCCTCTC

CTCTCCCCTTGCTCGCGCGTTCCCTCTCTTCCTCCGTTTTCTGGTGTGCT

GGAACGTTCAGCGGAATATGATGAATGATCACCTGTCACAGCTTGTTTAT

TATAATGCAGGCAATCAATTACACATCCCCAATGCTGGCCGGCCCGCAGG

AAATTTATATGCTCAGCACAAACCAATGTGAAAATGGAATCTCATTTGCC

AAATGTCTTTCTCCCCGTACAGCACGATGATTACAGTCTGTGTTTGTTTC

AACAGTCGTGTACAACTGACAGTGCCATCATTTACTGCCTGGCTCAGGTC

ACGTTACTCTAAGGCTTTATTTATGGTGTTACGAAGGGCAGCACAGGAAA

AGGACAAGGGTGTCTGTCAGGGATGGCACTGTGTTAAAAAGTGGGCGTGC

AAGGGCCGCATTCCCGGGCAGCCGCTGCAACCTCAGCCCCTGGGCCCTTA

CCTCCGCAGCCTCTCCCAGCATCCAGCTACCCAGACTCCAAGGCCCCAGG

CGAGAGCCAGCTCTCGGTACCTGGAGCTCGGAGATATTGTATGGTAGTCC

TGACTATAAAGGTGGATCATATGTCGCCGTTCGAGATTTATAGCCGGGCG

CGGTTATATCCCGGCGAACGGTCGCAAGGTCCACCAGATGGTACTATCAT

TCAATGTCTCCTCTGGGATGTCAGAGGTGTTGATGAATGTTAGTTCCCGG

GACTTCGGAAAGAGGTCCCGTTGGAAGAGATGTGAATTGGAATTCACACC

CTATATTAAAATCTCCTCCAATCTTCACCTCTGAGACATGGCTGTCTCAA

GACTGTTTTGTTTCCCTTCCTGGTGGAATTTTGCACTTTTATGTCCTGTG

TAGCAGCAGGTAGTGTGGCTTTGAGAAAATAAAATGGCCACCTTGCTCCG

CTGTTCTTTCTTTGTAAAAAAAAAAAAAAAAAAAAAAAAACGGCATAGCG

GGCGTACTGGTGCTATGGATCGGAGACTGGGCCTTCAATACCCTTGCGCA

CTTGACATTTCAAGCCTATGCCCTGGCATTTCTTGCCACTCACCTAACCA

CCGAGCAACGCCCTTCAACCGCAACGTCTTCATCAAAGACTTTAAGA
