# Supplementary material for: Ancient exapted transposable elements promote nuclear enrichment of human long noncoding RNAs
Source: Genome Res. 2019 Feb;29(2):208–22. doi: 10.1101/gr.229922.117 (PMC6360812; doi:10.1101/gr.229922.117)
Supplement: Supplemental Material [file supp_gr.229922.117_Supplemental_File_S6.docx]

**Summary of evolutionarily conserved element annotations**

All BED-format files were originally obtained in coordinates of genome build hg19, and converted to hg38 using *LiftOver*.

|  | Number of elements | Average length (bp) | Source |
| --- | --- | --- | --- |
| Evolutionarily-conserved structures (ECS) | 3,275,619 | 149 | Smith et al (23847102) |
| PhastCons 46-Way: Vertebrate | 5,162,677 | 29 | UCSC Table Browser |
| PhastCons 46-Way: Placental Mammal | 3,743,161 | 32 | UCSC Table Browser |
| PhastCons 46-Way: Primate | 725,585 | 149 | UCSC Table Browser |
