# Supplementary material for: Ancient exapted transposable elements promote nuclear enrichment of human long noncoding RNAs
Source: Genome Res. 2019 Feb;29(2):208–22. doi: 10.1101/gr.229922.117 (PMC6360812; doi:10.1101/gr.229922.117)

**qRT-PCR Primers**

**Oligonucleotide Sequences**

Candidate LncRNA Primers

| Primer | Sequence |
| --- | --- |
| RP11-5407.1_Wild type_endogenous_F | CCCCTGGAGCAGAGATGAG |
| RP11-5407.1_Wild type_endogenous_R | GATGCCAATGTCAAATGCCAC |
| RP11-5407.1_Wild type_transfected_F* | GAGACCCAAGCTGGCTAGCGTG |
| RP11-5407.1_Wild type_transfected_R | GTCCCACCTCCTTCACAGGTG |
| RP11-5407.1_Mutant_transfected_F* | GAGACCCAAGCTGGCTAGCGTG |
| RP11-5407.1_Mutant_transfected_R | GACGGACTTTCCTCACAGCC |
| LINC00173_Wild type_endogenous_F | GCTACTGTCTAATTGCGTGC |
| LINC00173_Wild type_endogenous_R | CAGCCATGTCTCAGAGGTGA |
| LINC00173_Wild type_transfected_F* | CGGCATAGCAATCTTGGCC |
| LINC00173_Wild type_transfected_R | CCCTCTAGACTCGAGAACATC |
| LINC00173_Mutant_transfected_F | GGTGGATCATATGTCGCCG |
| LINC00173_Mutant_transfected_R | CAGCCATGTCTCAGAGGTGA |
| RP4-806M20.4_Wild type_endogenous_F | GCACTGGGGAATGGTGAACT |
| RP4-806M20.4_Wild type_endogenous_R | CGTAACAATCCGCAAGGCAG |
| RP4-806M20.4_Wild type_transfected_F* | CCTCACTGACGCCTCCTCG |
| RP4-806M20.4_Wild type_transfected_R | CCCTCTAGACTCGAGCACTC |
| RP4-806M20.4_Mutant_transfected_F | GCCGGGAGTTTTACCTACCT |
| RP4-806M20.4_Mutant_transfected_R | CCACTCAGCTGGAGAGAAGG |

* Primers designed against a transcribed region of the expression vector backbone

Control Gene Primers

| Primer | Sequence |
| --- | --- |
| MALAT1_F | GATTGAGGCGTTTTCCAAGA |
| MALAT1_R | ACTTTCTCCCCCAACTGCTT |
| GAPDH_F | TGGAAGGACTCATGACCAC |
| GAPDH_R | CCATCACGCCACAGTTTCC |

**Primer efficiency estimation**

|  | Amplification efficiency : E = 10^(-1/slope) | | |
| --- | --- | --- | --- |
|  | % efficiency = (E-1)*100% | |  |
| Primer | slope (m) | E | Efficiency (%) |
| RP11-5407.1_Wild type Endogeneous | -3.16 | 1.072314 | 107.23 |
| RP11-5407.1_Wild type Transfected | -4.12 | 1.748713 | 74.87126 |
| RP11-5407.1_Mutant Transfected | -3.78 | 1.838867 | 83.88668 |
| LINC00173_Wild type Endogeneous | -3.662 | 0.875317 | 87.53175 |
| LINC00173_Wild type Transfected | -3.43 | 1.956794 | 95.67944 |
| LINC00173_Mutant Transfected | -3.844 | 1.820311 | 82.03114 |
| RP4-806M20.4_Wild type Endogeneous | -4.173 | 0.736343 | 73.63439 |
| RP4-806M20.4_Wild type Transfected | -4.115 | 1.749901 | 74.99005 |
| RP4-806M20.4_Mutant Transfected | -3.488 | 1.935073 | 93.50725 |


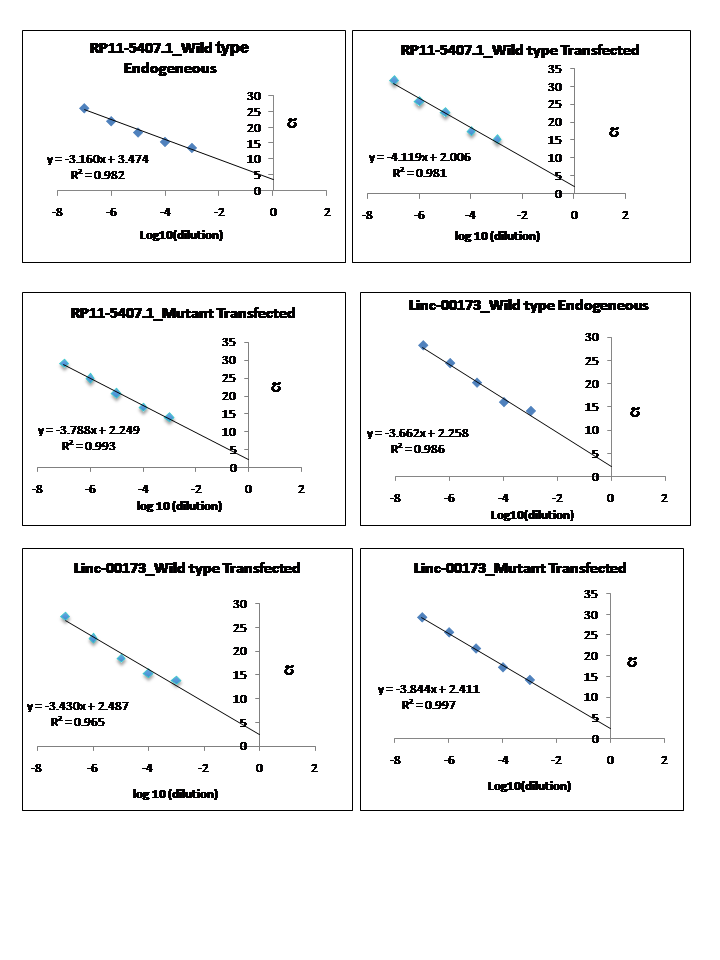

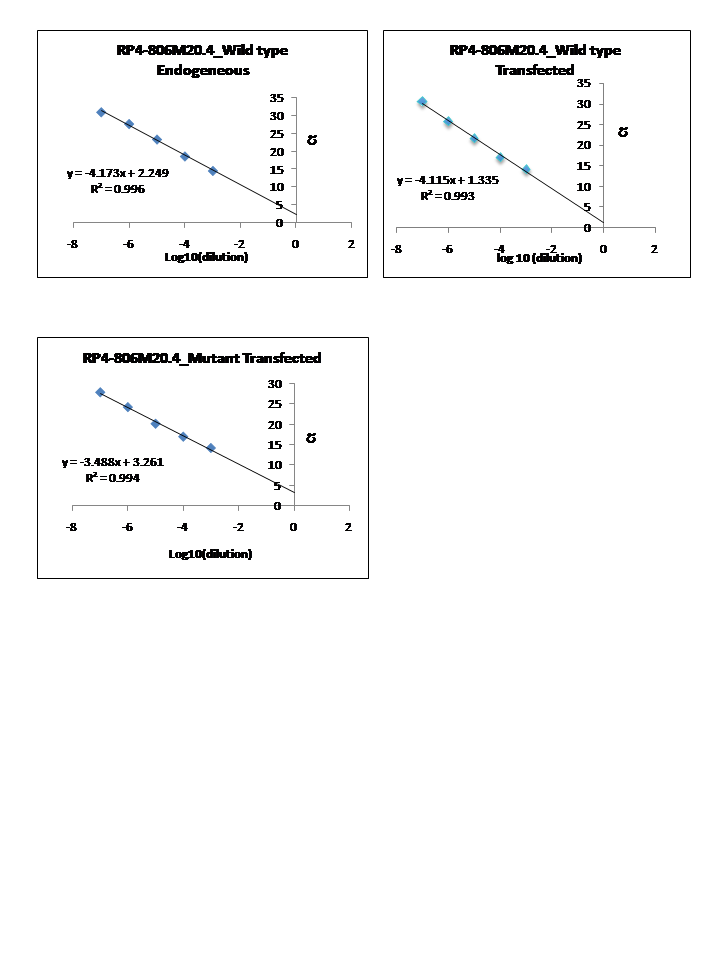

Supplement: Supplemental Material [file supp_gr.229922.117_Supplemental_File_S7.docx]
