# Supplementary material for: Ancient exapted transposable elements promote nuclear enrichment of human long noncoding RNAs
Source: Genome Res. 2019 Feb;29(2):208–22. doi: 10.1101/gr.229922.117 (PMC6360812; doi:10.1101/gr.229922.117)
Supplement: Supplemental Material [file supp_gr.229922.117_Supplemental_Legends.pdf]

Supplemental File S1: RIDL Annotation. File is in BED format with coordinates for human genome assembly GRCh38 / hg38. It was created by merging a subset of the exonic TEs from Supplemental File S5, and thus some lines may be composed of merged features.

The 4th "name" column contains information on the TE (modified from *RepeatMasker* annotation) in comma-separated format:

(positive genomic strand) Chromosome of TE annotation, start of TE annotation, end of TE annotation, strand of TE annotation, TE name, TE class, TE family, begin in repeat, end in repeat, -(left in repeat), hypothetical start if TE were full length, hypothetical end if TE were full length.

(negative genomic strand) Chromosome of TE annotation, start of TE annotation, end of TE annotation, strand of TE annotation, TE name, TE class, TE family, , -(left in repeat), end in repeat, begin in repeat, hypothetical start if TE were full length, hypothetical end if TE were full length.

Supplemental File S2: Insertion profiles. PDF images of insertion profiles of all RIDLs (blue) and intronic insertions (red). *x* axis shows the entire consensus sequence of a given TE type. *y* axis indicates the frequency with which each nucleotide position is present in the aggregate of all insertions. "CC": Spearman correlation coefficient of the two profiles. "Data" / "Reference" indicate the numbers of individual insertions considered for RIDLs / intronic insertions, respectively.

Supplemental File S3: RIDL-lncRNAs. List of GENCODE v21 lncRNAs carrying one or more RIDLs. First column contains gene ID and second column the number of RIDLs present in the lncRNA.

Supplemental File S4: Wild-type and mutant synthesized plasmid sequences. Coloured sequences indicate wild-type / scrambled RIDL sequences.

Supplemental File S5: Exonic Transposable Element Annotation. File is in BED format with coordinates for human genome assembly GRCh38 / hg38. The file contains the coordinates of genomic nucleotides that overlap both TEs and merged exons of lncRNA genes.

The 4th "name" column contains information on the TE (from *RepeatMasker* annotation) in comma-separated format:

Chromosome of TE annotation, start of TE annotation, end of TE annotation, strand of TE annotation, TE name, TE class, TE family, left in repeat, end in repeat, begin in repeat, hypothetical start if TE were full length, hypothetical end if TE were full length.

In the 5th "score" column is indicated the TE classification:

+1 : tss.plus ; +2 : splice_acc.plus ; +3 : encompass.plus ; +4 : splice_don.plus; +5 : tts.plus ; +6 : inside.plus. The sign (+-) denotes whether the annotated strand of the TE lies on the same/opposite strand with respect to that of the overlapping lncRNA.

Supplemental File S6: Summary of evolutionary conservation data used

Supplemental File S7: Primer sequences and amplification efficiencies
